# Disruption of PPARG Activity and CPT1A Regulation by Bisphenol A: Implications for Hepatic Lipid Metabolism

**DOI:** 10.1111/jcmm.70416

**Published:** 2025-05-09

**Authors:** Xiliang Zhu, Qi Liu, Zhaoyun Cheng, Yi Luo

**Affiliations:** ^1^ Department of Cardiovascular Surgery, Central China Subcenter of National Center for Cardiovascular Diseases, Henan Cardiovascular Disease Center, Fuwai Central‐China Cardiovascular Hospital Central China Fuwai Hospital of Zhengzhou University Zhengzhou China; ^2^ Department of Cardiology, Sir Run Run Shaw Hospital, School of Medicine Zhejiang University Hangzhou China; ^3^ Department of Cardiology, The Affiliated Hospital Key Laboratory of Medical Electrophysiology of the Ministry of Education, Medical Electrophysiological Key Laboratory of Sichuan Province, Institute of Cardiovascular Research Southwest Medical University Luzhou China

**Keywords:** bisphenol a (BPA), endocrine disruption, hepatic lipid metabolism, PPARG, transcriptomic analysis

## Abstract

Bisphenol A (BPA) is a widely used industrial chemical with potential endocrine‐disrupting effects on metabolic processes. This study investigates the impact of BPA on hepatic function and transcriptional regulation in mouse livers and AML12 cells. Male mice were exposed to low (5 g/kg) and high (50 g/kg) doses of BPA for six weeks. Transcriptomic analysis was performed on liver tissues, and histological examinations were conducted. AML12 cells were treated with varying BPA concentrations, and PPARG transcriptional activity was assessed using a luciferase reporter assay. Additionally, molecular docking, molecular dynamics (MD) simulations, drug affinity responsive target stability (DARTS), cellular thermal shift assay (CETSA), MM‐PBSA calculations, and multi‐species protein structure comparative analysis were employed to analyse the interaction between BPA and PPARG. Transcriptomic analysis revealed a decrease in differentially expressed genes with higher BPA doses, with low‐dose exposure significantly downregulating hepatic *Cpt1a* mRNA levels. Histological examination indicated lipid vacuole formation at high doses without collagen deposition. BPA consistently inhibited PPARG activity in both MCF7 cells and mouse livers. BPA exposure disrupts hepatic lipid metabolism and PPARG activity, highlighting its role as an endocrine disruptor. Further research is needed to elucidate the long‐term effects of BPA on liver health.

## 1 Introduction

Bisphenol A (BPA) is a widespread endocrine disruptor present in various environmental matrices, raising significant health concerns due to its potential interference with hormonal signalling pathways [1, 2, 3, 4, 5]. Its ability to bind multiple nuclear receptors indicates a complex mechanism affecting gene expression and metabolic processes, particularly in lipid metabolism [5, 6, 7, 8, 9]. Among the regulatory proteins, PPARG (peroxisome proliferator‐activated receptor) is a crucial transcription factor that orchestrates lipid metabolism in the liver and adipose tissue [10, 11, 12]. Specifically, PPARG modulates the expression of genes involved in fatty acid uptake, transport, and oxidation, thus regulating hepatic lipid homeostasis. In adipose tissue, PPARG is indispensable for adipogenesis, facilitating the differentiation of preadipocytes into mature adipocytes and governing processes such as lipid storage, mobilisation, and catabolism. Through these functions, PPARG maintains a finely tuned balance of lipid metabolism across both liver and adipose tissues, ultimately contributing to systemic energy homeostasis and metabolic health. Disruption of PPARG function by BPA could profoundly impact metabolic homeostasis, yet the dynamic interactions between BPA and PPARG remain largely unexplored.

CPT1A, a key gene in fatty acid β‐oxidation, is essential for lipid metabolism regulation [13, 14, 15]. Variations in CPT1A levels can lead to metabolic disturbances, emphasising the need to understand the regulatory mechanisms governing its expression [16]. Additionally, CPT1A is closely intertwined with PPARG, a pivotal transcription factor responsible for maintaining hepatic and adipose lipid homeostasis. PPARG activation modulates CPT1A expression and activity, thereby influencing overall fatty acid β‐oxidation and systemic energy balance. The potential for BPA to modulate CPT1A expression via PPARG raises critical questions about BPA's impact on lipid metabolism.

This study aims to elucidate the effects of BPA on PPARG and its subsequent influence on hepatic lipid metabolism. We will analyse the interactions between BPA and PPARG and investigate the susceptibility of PPARG homologues across species to BPA exposure, providing insights into the broader implications of BPA's effects on metabolic regulation.

## 2 Materials and Methods

### 2.1 Animal Study and Cell Culture

In this study, male C57BL/6N mice (8 weeks old) from SLAC (Shanghai, China) were used to assess the effects of varying doses of Bisphenol A (BPA, B108652, ≥ 99% (GC); Aladdin, Shanghai, China) on hepatic function. Mice were divided into three groups (*n* = 10) and fed standard chow, chow with a low BPA dose (5 g/kg), or chow with a high BPA dose (50 g/kg) for six weeks under controlled conditions. The 5 g/kg dose was selected to reflect a moderate, subchronic exposure level, enabling the detection of early or subtle biochemical and histological alterations that might be missed at lower concentrations. In contrast, the 50 g/kg dose was chosen to model a high‐challenge scenario for assessing potential severe or overt hepatotoxic effects as well as to examine the broader toxicological spectrum of BPA. Weekly monitoring of body weight and health was conducted, and at the end of the treatment, liver tissues were collected for biochemical assays following ethical guidelines.

Additionally, AML12 cells (derived from normal 3‐month‐old mouse hepatocytes; ATCC, Manassas, VA, USA) were cultured in DMEM/F‐12 medium supplemented with dexamethasone and insulin‐transferrin‐selenium‐pyruvate. After seeding (2×10^5 cells/well in 6‐well plates), cells were treated with BPA stock solutions (prepared in DMSO) at final concentrations of 1 μM (low dose) or 20 μM (high dose) for 24 h. The final concentration of DMSO in all treatments was maintained at 0.1%. The effects on PPARG were evaluated under various conditions, including vehicle control and treatments with GW1929, a PPARG agonist. Cells were then harvested for assays to assess PPARG activity.

### 2.2 Quantitative Analysis of Lipid Accumulation Using Oil Red O Staining and BODIPY 493/503

AML12 cells from ATCC were cultured in DMEM/F‐12 medium supplemented with 40 ng/mL dexamethasone and 1% insulin‐transferrin‐selenium‐pyruvate(C0342; Beyotime, Shanghai, China), maintained at 37°C in a 5% CO₂ humidified atmosphere. Cells were seeded at a density of 1×10^5 cells per well in 6‐well plates for overnight adherence. Following treatment with vehicle control (DMSO, S24295;Yuanye, Shanghai, China), 1 μM BPA, 10 μM GW1929 (HY‐15655; MCE, Shanghai, China) or a combination of 1 μM BPA and 10 μM GW1929 for 24 h, cells were washed with phosphate‐buffered saline (PBS) and fixed in 10% formalin for 30 min. For lipid accumulation analysis, oil red O staining was performed by incubating cells with oil red O solution (R32699, Yuanye) for 15 min, followed by excess stain removal and visualisation under a microscope. For quantitative assessment, stained droplets were dissolved in 100% isopropanol, and optical density (OD) was measured at 500 nm using a spectrophotometer (Thermo Fisher Scientific, Waltham, MA, USA). Additionally, lipid droplets were stained with 1 μg/mL BODIPY 493/503(HY‐W090090; MCE, Shanghai, China) in PBS for 30 min, protected from light, followed by washing with PBS. Stained lipid droplets were visualised and imaged using a fluorescence microscope (Leica Microsystems, Wetzlar, Germany), with fluorescence intensity providing a quantitative measure of lipid accumulation under different treatment conditions.

### 2.3 Liver Histological Analysis

Liver tissues were collected from mice post‐treatment and fixed in 10% neutral‐buffered formalin before being embedded in paraffin. Serial sections of 5 μm thickness were prepared using a microtome and mounted on glass slides. The sections underwent deparaffinisation in xylene and rehydration through a graded series of ethanol. Haematoxylin and eosin (H&E) staining was performed to visualise the tissue architecture and lipid droplets, followed by quantification of lipid droplet vacuole areas using ImageJ software (NIH, Bethesda, MD, USA).

Additionally, Sirius red staining was conducted to assess collagen deposition and fibrosis, with sections incubated in Weigert's iron haematoxylin for nuclear staining and subsequently stained in Sirius red solution. Fibrosis quantification was also performed using ImageJ software to measure fibrotic areas.

For lipid accumulation analysis, liver tissues were harvested, fixed, and embedded in the optimal cutting temperature (OCT) compound (Sakura Finetek, Torrance, CA, USA). Frozen sections of 10 μm thickness were stained with oil red O solution, counterstained with haematoxylin, and analysed under a light microscope. Quantitative analysis of lipid droplet areas was conducted using ImageJ software, providing comprehensive insights into lipid accumulation and fibrosis in liver tissues under various experimental conditions.

### 2.4 Analysis of PPARG Expression and Localization in Liver and AML12 Cells

Liver tissues and AML12 cells underwent immunofluorescence staining to assess PPARG expression and localization. Liver sections were incubated overnight at 4°C with primary antibodies against PPARG (1:200, AF6284; Affinity). After PBS washes, sections were treated with fluorophore‐conjugated secondary antibodies (1:500, S0018; Affinity, Suzhou, China) for 1 h at room temperature, followed by DAPI counterstaining. Fluorescence images were captured using a Leica DM6 B microscope at 20× magnification (Wetzlar, Hesse, Germany). For AML12 cells, after treatment and fixation, cells were permeabilized with 0.2% Triton X‐100, blocked with 5% goat serum, and incubated overnight with primary antibodies. Quantitative analysis of PPARG expression was performed using ImageJ software.

### 2.5 qPCR Analysis of Gene Expression in PPARG‐Overexpressing AML12 Cells

To evaluate the impact of PPARG overexpression on lipid metabolism‐related gene expression in AML12 cells, quantitative PCR (qPCR) analysis was performed. AML12 cells were transfected with a PPARG overexpression plasmid using Lipofectamine 3000, following the manufacturer's instructions. After 48 h, total RNA was extracted using the RNeasy Mini Kit. The RNA concentration was measured with a NanoDrop 2000 spectrophotometer (Thermo Fisher Scientific, Waltham, MA, USA) and subsequently reverse‐transcribed into cDNA using the High‐Capacity cDNA Reverse Transcription Kit (Applied Biosystems, Foster City, CA, USA). qPCR was conducted with PowerUp SYBR Green Master Mix on a QuantStudio 3 Real‐Time PCR System (Thermo Fisher Scientific, Waltham, MA, USA). Primers for target genes, including *Acox1*, *Scp2*, *Acadm*, *Acaa1*, *Acadl*, *Cpt1a*, *Cpt1b*, and *Cpt1c*, were designed using Primer‐BLAST and synthesised by Sangon Technologies (Shanghai, China). The qPCR protocol involved an initial denaturation at 95°C for 10 min, followed by 40 cycles of 95°C for 15 s and 60°C for 1 min. Relative gene expression levels were normalised to the housekeeping gene *Gapdh* and calculated using the 2^‐ΔΔ^C^_t_ method.

### 2.6 PPARG Activity and Transcriptional Regulation of CPT1A

AML12 cells were cultured in DMEM/F‐12 medium with dexamethasone and insulin‐transferrin‐selenium‐pyruvate at 37°C with 5% CO₂. Cells were seeded in 24‐well plates and transfected with the PPARG Luciferase Reporter Plasmid (YB081B; Ybio) using Lipofectamine 3000. After 24 h, cells were treated with 1 μM or 20 μM BPA, or DMSO as a control, along with additional treatments including GW1929 (10 μM) and combinations with BPA. Following treatment, cells were lysed, and luciferase activity was measured using the Dual‐Luciferase Reporter Assay System, normalising with *Renilla* luciferase activity to assess PPARG transcriptional activity.

Additionally, sequences of the CPT1A promoter region (2000 bp upstream) for mouse were retrieved from the NCBI database. Motif analysis identified PPARG binding sites, and wide‐type, mutated, and deleted luciferase reporter constructs(Baimaike, Beijing, China) were generated to evaluate CPT1A transcriptional activity under conditions of PPARG co‐overexpression and various treatments in AML12 cells.

### 2.7 Structural Prediction of PPARG Binding to the Cpt1a Promoter

To predict the binding conformation of bovine PPARG to the CPT1A promoter, we employed the AlphaFold3 server. The bovine PPARG protein sequence was sourced from the UniProt database, while the CPT1A promoter DNA sequence containing the PPARG binding motif was obtained from the Ensembl genome database. Both sequences were uploaded to AlphaFold3, targeting the interaction between bovine PPARG and the binding motif for structural prediction. The server utilised its deep learning algorithm to generate a predicted 3D conformation of the PPARG‐DNA complex, which was then visualised using PyMOL (v2.6). Structural analysis focused on identifying key residues involved in the interaction, examining the binding interface, hydrogen bonds, and hydrophobic interactions to elucidate the molecular mechanisms by which PPARG regulates CPT1A transcription.

### 2.8 Western Blot Analysis of PPARG and CPT1A Protein Levels Under Different Treatment Conditions

To evaluate the changes in PPARG and CPT1A protein levels under various treatment conditions (VEH, VEH + BPA, GW1929, and GW1929 + BPA), western blot analysis was performed. AML12 cells were cultured in DMEM supplemented with 10% FBS and 1% penicillin–streptomycin and treated with the respective conditions for 24 h. Following treatment, cells were lysed using RIPA buffer supplemented with protease and phosphatase inhibitors. Protein concentration was determined using the BCA protein assay kit (P0009; Beyotime). Equal amounts of protein (30 μg) from each sample were separated by SDS‐PAGE on a 10% gel and transferred onto PVDF membranes. The membranes were blocked with 5% non‐fat dry milk in TBS‐T for 1 h at room temperature. Subsequently, the membranes were incubated overnight at 4°C with primary antibodies against PPARG and CPT1A. After washing with TBS‐T, the membranes were incubated with HRP‐conjugated secondary antibodies for 1 h at room temperature. Protein bands were visualised using an enhanced chemiluminescence detection system and imaged with the ChemiDoc MP imaging system. Densitometric analysis was performed using ImageJ software, and protein levels were normalised to GAPDH as a loading control.

### 2.9 Assessment of Plasma Biochemical Markers and Hepatic Lipid Content

Plasma biochemical markers, including alanine aminotransferase (ALT), aspartate aminotransferase (AST), alkaline phosphatase (ALP), albumin (ALB), total protein (TP), total cholesterol (TC), high‐density lipoprotein cholesterol (HDL‐C), low‐density lipoprotein cholesterol (LDL‐C), and triglycerides (TG) were quantitatively measured using a fully automated biochemical analyser (Hitachi 71,800, Tokyo, Japan) with assay kits from BIOSINO Biotechnology (Beijing, China). For liver lipid content analysis, liver tissues were weighed and homogenised, and lipids were extracted using a methanol‐chloroform mixture (2:1, v/v). The solvent was evaporated using a vacuum centrifugal concentrator, and the remaining lipids were dissolved in a 10% Triton X‐100 solution. Total cholesterol and triglycerides were quantified using specific assay kits from APPLYGEN (Beijing, China; #E1015 for TC and #E1013 for TG) on an EnVision 2105 Multimode Plate Reader (PerkinElmer, Fremont, CA, USA), following the manufacturer's protocols. The concentrations of TC and TG in liver samples were expressed as μmol per gram of liver tissue.

### 2.10 Bioinformatics Analysis of BPA‐Exposed Transcriptome Data

Gene expression data for mouse livers exposed to varying doses of BPA were obtained from the GEO database (accession number GSE26728). Differentially expressed genes (DEGs) were identified using the limma package, applying a linear model to the expression data with empirical Bayes moderation for standard error adjustment. DEGs were defined as those with an adjusted *p*‐value < 0.05 and an absolute log_2_ fold change > 1. Pathway enrichment analysis of the identified DEGs was performed using the clusterProfiler package, with KEGG pathways as the reference database, considering results significant at an adjusted *p*‐value < 0.05. To evaluate transcription factor activity under different BPA exposure conditions, GSVA and ssGSEA algorithms were employed, sourcing gene sets from MSigDB. Transcriptome data for MCF7 cells exposed to different BPA doses were retrieved from the GEO database (accession number GSE211183) and processed using the DESeq2 package for normalisation. Transcription factor regulons were calculated using the RTN package, which involved reconstructing transcriptional regulatory networks. The activity changes of various transcription factors under different BPA concentrations were assessed using the aREA method within the RTN package (Bioconductor, Seattle, WA, USA), and a correlation matrix of transcription factor activities was computed to identify potential co‐regulatory relationships, with significant correlations (*p*‐value < 0.05) used to infer these relationships.

### 2.11 Molecular Docking and Molecular Dynamics Simulations

Molecular docking and analysis of PPARG with BPA were performed using the Protein Data Bank and Biopython (Open Source, http://www.biopython.org, Cambridge, UK) for preprocessing. BPA structures were retrieved from PubChem and converted with OpenBabel, while the active site of human PPARG was defined using Dogscorer. Homologous proteins were identified via Foldseek, and structures were sourced from the AlphaFold database. Molecular docking was executed with AutoDock Vina, eliminating redundant structures based on an RMSD cutoff of 0.19. For molecular dynamics simulations, both ligand‐free PPARG (PPARG‐Apo) and BPA‐bound PPARG (PPARG‐BPA) were prepared from the human PPARG structure (PDB ID: 2hfp) and solvated in a TIP3P water box. After energy minimization and equilibration, 100 ns production simulations were conducted under periodic boundary conditions. Analyses for RMSD, RMSF, and radius of gyration were performed using GROMACS, while the secondary structure content was assessed with DSSP, and binding interactions were evaluated using MM‐PBSA calculations. Visualisation of docking results and molecular dynamics trajectories was conducted using PyMOL (Schrödinger LLC, New York, NY, USA) and VMD (v1.9.3) (University of Illinois at Urbana‐Champaign, Urbana, IL, USA).

## 3 Results

### 3.1 Transcriptomic Changes in Mouse Livers Exposed to Different BPA Doses

**FIGURE 1:**
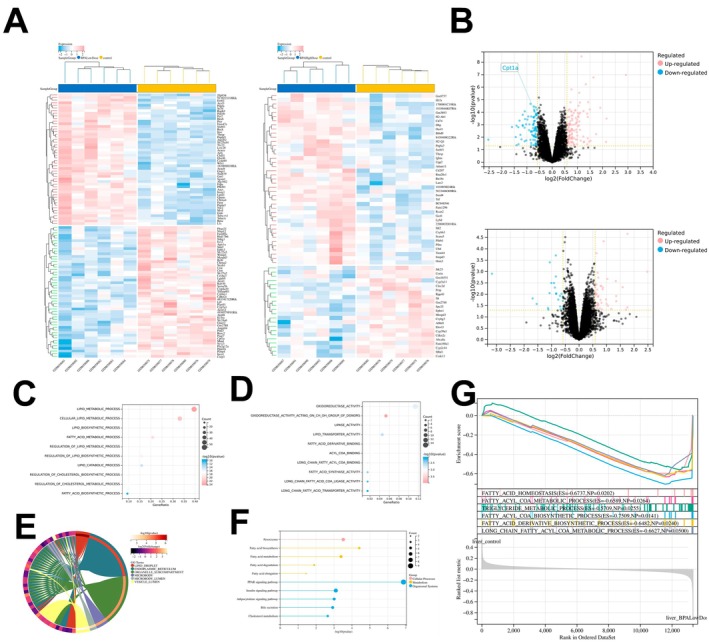
Transcriptomic changes in the livers of mice exposed to different doses of BPA. (A) Heatmap of differentially expressed genes (DEGs) in the livers of mice exposed to low‐dose (left) and high‐dose (right) BPA, showing distinct expression patterns. (B) Volcano plots indicating the distribution of DEGs in response to low‐dose (top) and high‐dose (bottom) BPA exposure, highlighting significantly upregulated and downregulated genes. (C) Bubble plot of gene ontology (GO) biological processes enriched by DEGs in low‐dose BPA‐exposed livers, focusing on lipid metabolic processes. (D) Bubble plot of GO molecular functions enriched by DEGs in low‐dose BPA‐exposed livers, emphasising activities such as lipase activity and fatty acid synthesis. (E) Chord diagram illustrating the relationship between DEGs and lipid‐related cellular components in low‐dose BPA‐exposed livers, particularly lipid droplets. (F) KEGG pathway enrichment analysis showing the activation of pathways involved in fatty acid synthesis, metabolism, and degradation in low‐dose BPA‐exposed livers. (G) Gene set enrichment analysis (GSEA) indicating active signalling pathways related to hepatic fatty acid and triglyceride metabolism under low‐dose BPA exposure.

Analysis of hepatic transcriptomic changes in mice exposed to high and low doses of BPA revealed a reduced number of DEGs in the high‐dose group compared to the low‐dose group (Figure 1A,B). This suggests a potential non‐monotonic dose–response relationship, warranting further investigation into the mechanisms underlying this observation. Specifically, low‐dose BPA exposure significantly downregulated hepatic Cpt1a mRNA levels, while high‐dose exposure did not affect Cpt1a transcription (Figure 1B). Regardless of BPA dose, DEGs were enriched in pathways related to lipid metabolism and fatty acid metabolism and molecular functions associated with lipase activity, fatty acid synthesis, and lipid transport, indicating a disruption of hepatic lipid metabolic homeostasis by BPA (Figure 1C,D). DEGs in the low‐dose BPA group were significantly enriched in lipid droplets at the cellular component level (Figure 1E). KEGG pathway enrichment analysis revealed activation of fatty acid synthesis, metabolism, and degradation pathways, strongly linked to the PPAR signalling pathway (Figure 1F). Furthermore, GSEA analysis of the transcriptomic data confirmed the activation of fatty acid and triglyceride metabolism‐related signalling pathways under low‐dose BPA exposure (Figure 1G). These findings highlight the complex and potentially dose‐dependent effects of BPA on hepatic lipid metabolism. Further research is needed to elucidate the specific mechanisms by which BPA exerts these effects and to determine the long‐term consequences of BPA exposure on liver health.

### 3.2 BPA‐Induced Inhibition of Transcription Factor Activity in MCF7 Cells and Mouse Livers

**FIGURE 2:**
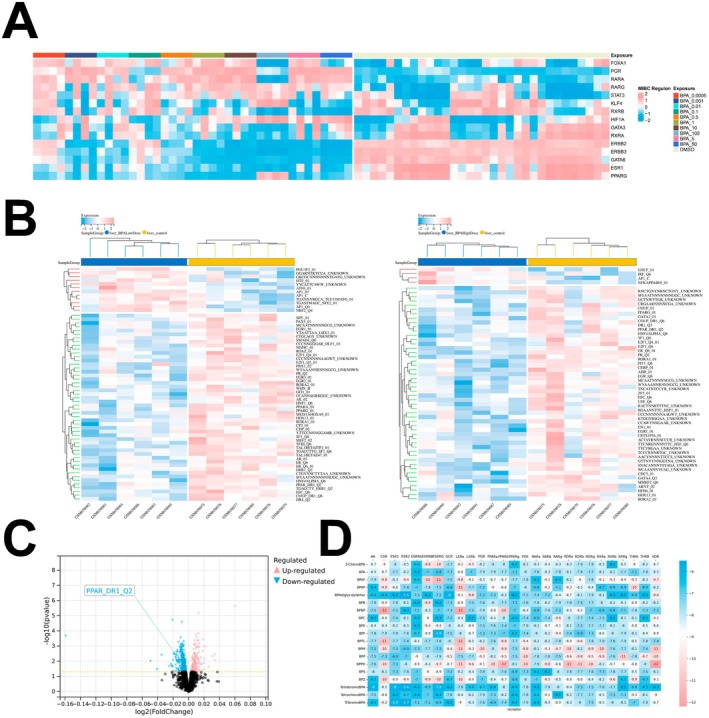
Effects of BPA on transcription factor activity in MCF7 breast cancer cells and mouse liver. (A) Heatmap showing changes in regulons of various transcription factors in MCF7 cells exposed to different concentrations of BPA (0.0005–100 μM), illustrating inhibition of PPARG activity at all concentrations except 100 μM. (B) Heatmaps depicting the results of GSVA analysis in mouse liver, indicating significant suppression of PPARG activity under both low‐dose (left) and high‐dose (right) BPA exposure, along with suppression of multiple transcription factors. (C) ssGSEA analysis displaying that low‐dose BPA exposure leads to the transcriptional repression of PPAR‐related receptors' regulons. (D) Molecular docking studies of common nuclear receptors with BPA and its derivatives, showing moderate affinity of BPA for PPARG.

We investigated the impact of BPA exposure (0.0005–100 μM) on the activity of various transcription factors in the MCF7 cell line, a well‐established model for assessing BPA toxicity due to its high oestrogen receptor expression (Figure 2A). Except at the highest concentration (100 μM), BPA consistently suppressed PPARG activity. This observation aligns with GSVA analysis of mouse liver samples, which demonstrated significant PPARG activity suppression at both low and intermediate BPA doses (Figure 2B). Notably, both gene set variation analysis (GSVA) and single‐sample gene set enrichment analysis (ssGSEA) revealed that a broader range of transcription factors exhibited suppressed activity following BPA exposure, suggesting pleiotropic effects of BPA on transcriptional regulation (Figure 2C). Specifically, ssGSEA analysis indicated transcriptional repression of PPAR‐related receptor regulons at low BPA doses. Finally, molecular docking studies of common nuclear receptors with BPA and its derivatives revealed moderate binding affinity of BPA for PPARG (Figure 2D). These findings collectively suggest that BPA exerts its effects, at least in part, through the modulation of multiple transcription factors, including the significant downregulation of PPARG activity, particularly at lower concentrations. Further research is needed to fully elucidate the complex interplay between BPA and the transcriptional regulatory network.

### 3.3 Histological and Biochemical Effects of BPA on Mouse Liver

**FIGURE 3:**
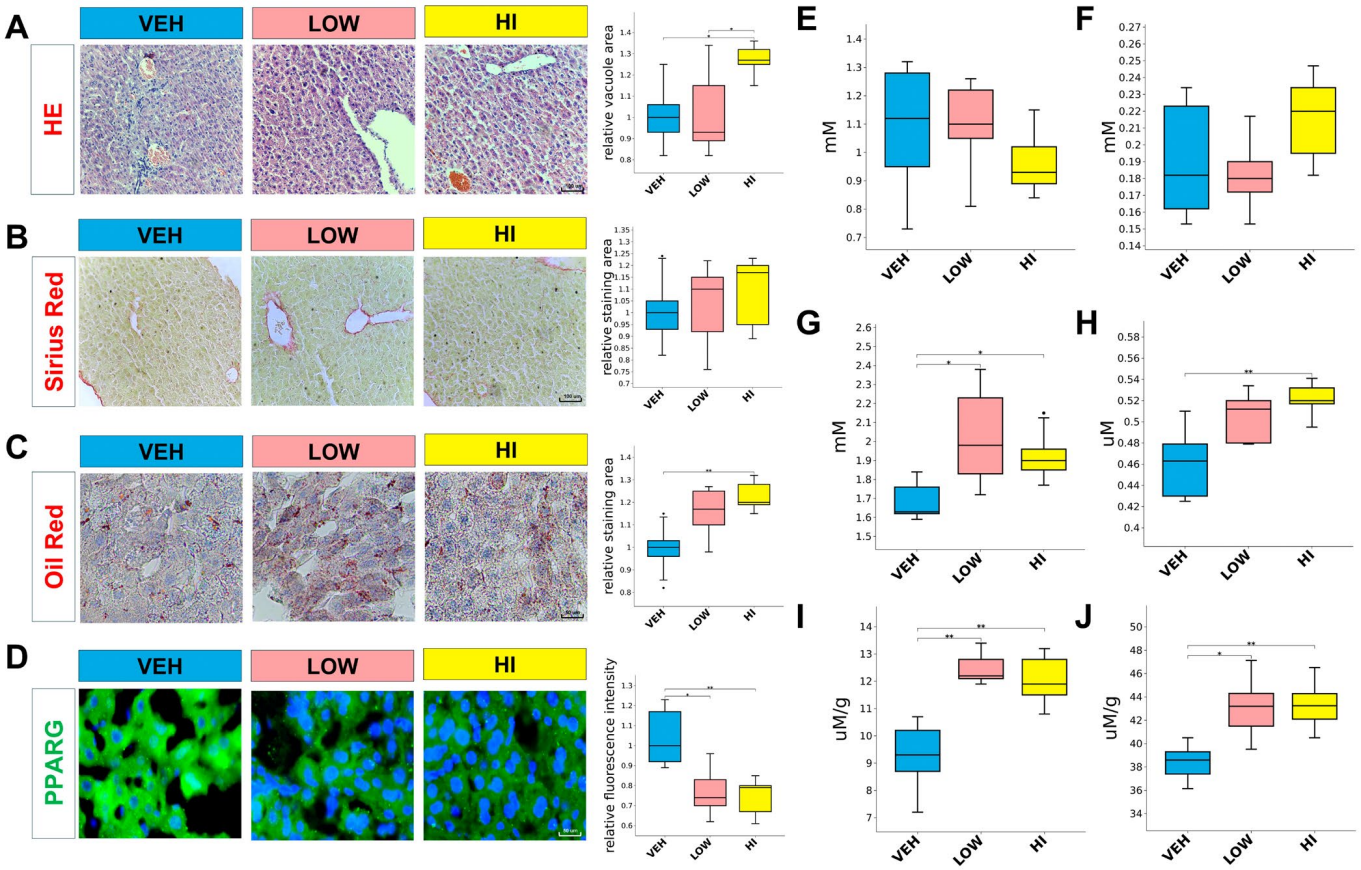
Histological and biochemical analyses of liver and serum samples under different treatments. (A) Representative haematoxylin and eosin (HE) staining images showing vacuole area in the liver under VEH, LOW, and HI treatments (left, 20×), with corresponding quantification (right). (B) Sirius red staining indicating collagen deposition in liver tissue across different treatments (left, 20×), with relative staining intensity quantified (right). (C) Oil Red O staining revealing lipid accumulation in liver sections for each treatment group (left, 40×), with relative staining intensity quantified (right). (D) Immunofluorescence staining of PPARG in liver tissues across VEH, LOW, and HI treatments (left, 40×), with quantification of relative fluorescence intensity (right). (E) Serum high‐density lipoprotein cholesterol (HDL‐C) levels measured in VEH, LOW, and HI groups. (F) Serum low‐density lipoprotein cholesterol (LDL‐C) levels across the different treatment groups. (G) Serum total cholesterol (TC) levels in VEH, LOW, and HI groups, with statistical significance indicated. (H) Serum total triglyceride (TG) levels for each treatment group, highlighting significant differences. (I) Hepatic total cholesterol (TC) content measured in VEH, LOW, and HI groups. (J) Hepatic total triglyceride (TG) content for VEH, LOW, and HI treatments, with statistical analysis. (Statistical significance: *p < 0.05, **p < 0.01, ***p < 0.001.)

Histological examination of mouse livers revealed no significant changes in architecture after low‐dose BPA exposure, while high‐dose exposure led to localised lipid‐vacuole‐like structures (Figure 3A). Sirius red staining indicated no collagen deposition in response to either dose (Figure 3B). Oil red O staining showed increased lipid accumulation following high‐dose BPA exposure (Figure 3C). Immunofluorescence analysis demonstrated reduced hepatic PPARG abundance in both exposure groups (Figure 3D). Additionally, there was no impact on high‐density lipoprotein cholesterol (HDL‐C) and low‐density lipoprotein cholesterol (LDL‐C) levels (Figure 3E,F). Liver function tests indicated no significant alterations, but both doses of BPA increased serum total cholesterol levels (Figures S1 and S3G), with only high‐dose exposure elevating serum triglycerides (Figure 3H). Consistent with serum profiles, both doses increased hepatic cholesterol and triglyceride accumulation (Figure 3I,J), suggesting a dose‐dependent effect of BPA on hepatic steatosis without overt fibrosis or significant liver dysfunction.

### 3.4 BPA's Effects on PPARG Activity, Lipid Metabolism, and Oxidative Stress in AML12 Cells

**FIGURE 4:**
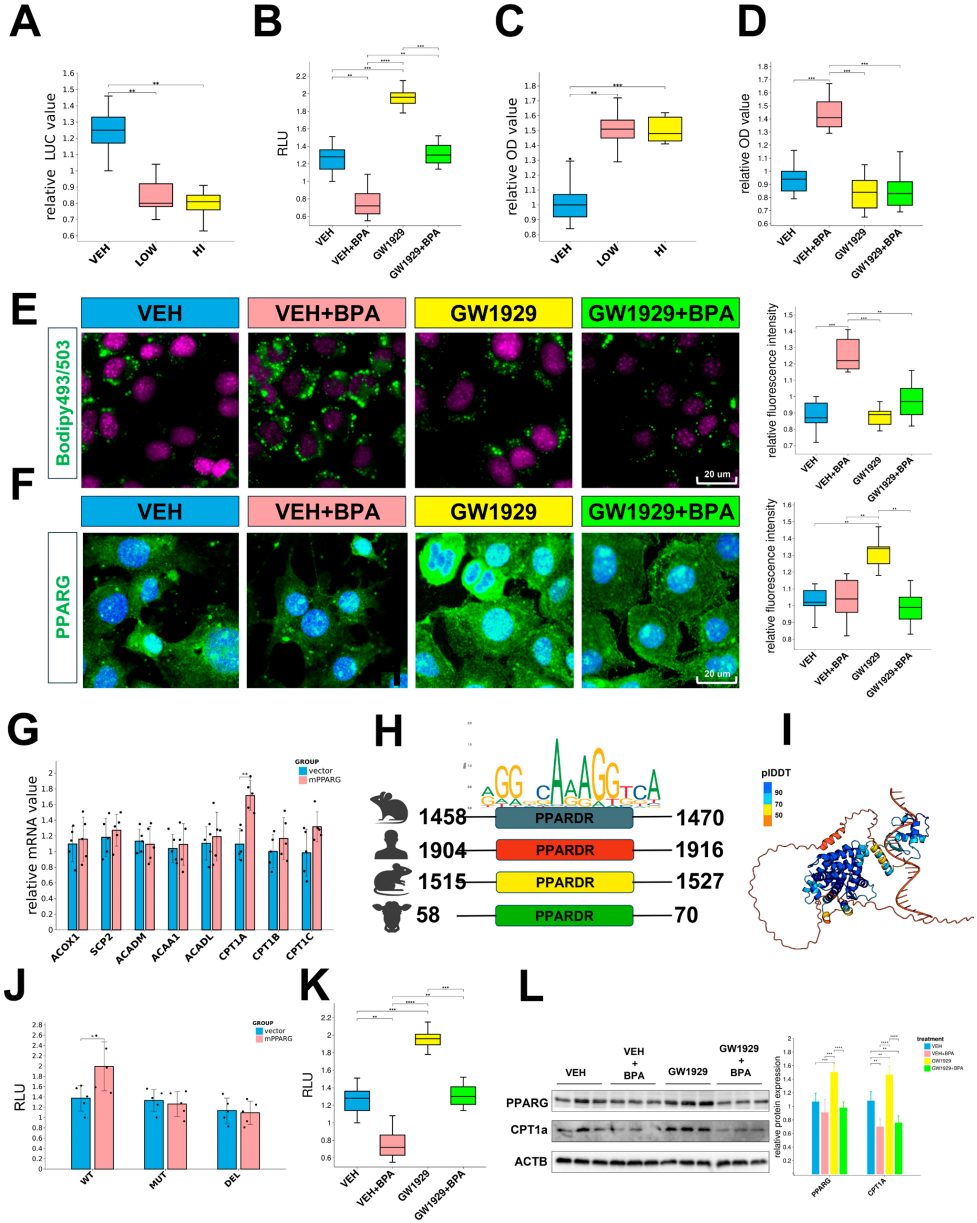
Effects of BPA and GW1929 on PPARG transcriptional activity and lipid accumulation in AML12 Cells. (A) Relative luciferase activity of PPARG in AML12 cells treated with VEH, LOW, and HI doses of BPA. (B) Relative luciferase activity of PPARG in AML12 cells treated with VEH, VEH + BPA, GW1929, and GW1929 + BPA. (C) Quantification of oil red O staining intensity indicating lipid accumulation in AML12 cells treated with VEH, LOW, and HI doses of BPA. (D) Quantification of oil red O staining intensity indicating lipid accumulation in AML12 cells treated with VEH, VEH + BPA, GW1929, and GW1929 + BPA. (E) Representative images of Bodipy 493/503 staining showing lipid droplet formation in AML12 cells treated with VEH, VEH + BPA, GW1929, and GW1929 + BPA (left, 40×), with corresponding quantification (right). (F) Representative immunofluorescence images of PPARG in AML12 cells across different treatment groups (left, 40×), with corresponding quantification (right). (G) Relative mRNA expression levels of genes associated with adipogenic differentiation and lipid droplet formation in AML12 cells under different treatments, including CPT1a. (H) Conserved PPARG binding motifs in the upstream promoter regions of CPT1a in mice, humans, rats, and cows. (I) Predicted binding interaction between PPARG and the PPARG binding site in the mouse CPT1a promoter region using Alphafold3. (J) Relative luciferase activity of wild‐type (WT), mutant (MUT), and deleted (DEL) PPARG binding site CPT1a reporter constructs in AML12 cells. (K) The effects of GW1929 and BPA on transcriptional activity in the wild‐type CPT1a reporter were examined. (L) Western blot analysis of PPARG and CPT1a protein levels in AML12 cells treated with VEH, BPA, GW1929, and GW1929 + BPA, with quantification (right). (Statistical significance: *p < 0.05, **p < 0.01, ***p < 0.001.)

Using a luciferase reporter assay, we demonstrated that both low and high doses of BPA significantly suppressed PPARG transcriptional activity in AML12 cells (Figure 4A). The PPAR agonist GW1929 enhanced PPARG transcriptional activity, an effect completely abolished by BPA co‐treatment (Figure 4B). BPA exposure at various concentrations increased oil red O staining intensity in AML12 cells, indicating enhanced lipid accumulation (Figure 4C). While GW1929 alone did not alter basal oil red O staining, it significantly attenuated the BPA‐induced increase in lipid accumulation (Figure 4D). Consistent with these findings, BPA exposure increased lipid droplet formation in AML12 cells, an effect reversed by GW1929 (Figure 4E). Furthermore, GW1929 promoted PPARG nuclear translocation, an effect also counteracted by BPA (Figure 4F).

Analysis of genes involved in adipogenesis and lipid droplet formation revealed that Cpt1a mRNA levels were significantly increased upon PPARG overexpression (Figure 4G). Sequence analysis of the upstream promoter regions of murine, human, rat, and bovine Cpt1a genes revealed conserved PPARG binding motifs (Figure 4H). AlphaFold3 modelling predicted the interaction between PPARG and the PPAR response element within the murine Cpt1a promoter (Figure 4I). Functional studies using Cpt1a reporter constructs containing wild‐type, mutated, and deleted PPAR response elements demonstrated that mutations or deletions significantly suppressed downstream transcriptional activity (Figure 4J). In AML12 cells stably expressing the wild‐type CPT1a reporter, BPA significantly attenuated GW1929‐mediated augmentation of transcriptional activity (Figure 4K). At the protein level, BPA similarly counteracted the GW1929‐mediated increase in PPARG and CPT1A abundance (Figure 4L). Considering that CPT1A localises to the mitochondrial membrane, we subsequently assessed the mitochondrial membrane potential in BPA‐exposed AML12 cells. Our findings showed that BPA reduced the mitochondrial membrane potential, and this effect was not rescued by PPARG activation(Figure S2A). In contrast, BPA exposure elevated reactive oxygen species (ROS) levels, whereas PPARG activation by GW1929 mitigated BPA‐induced oxidative stress in hepatocytes(Figure S2B).

These data strongly indicate that BPA disrupts PPARG‐mediated transcriptional regulation of Cpt1a, thereby contributing to its impacts on both lipid metabolism and oxidative stress.

### 3.5 BPA Docking and Interaction With PPARG A

**FIGURE 5:**
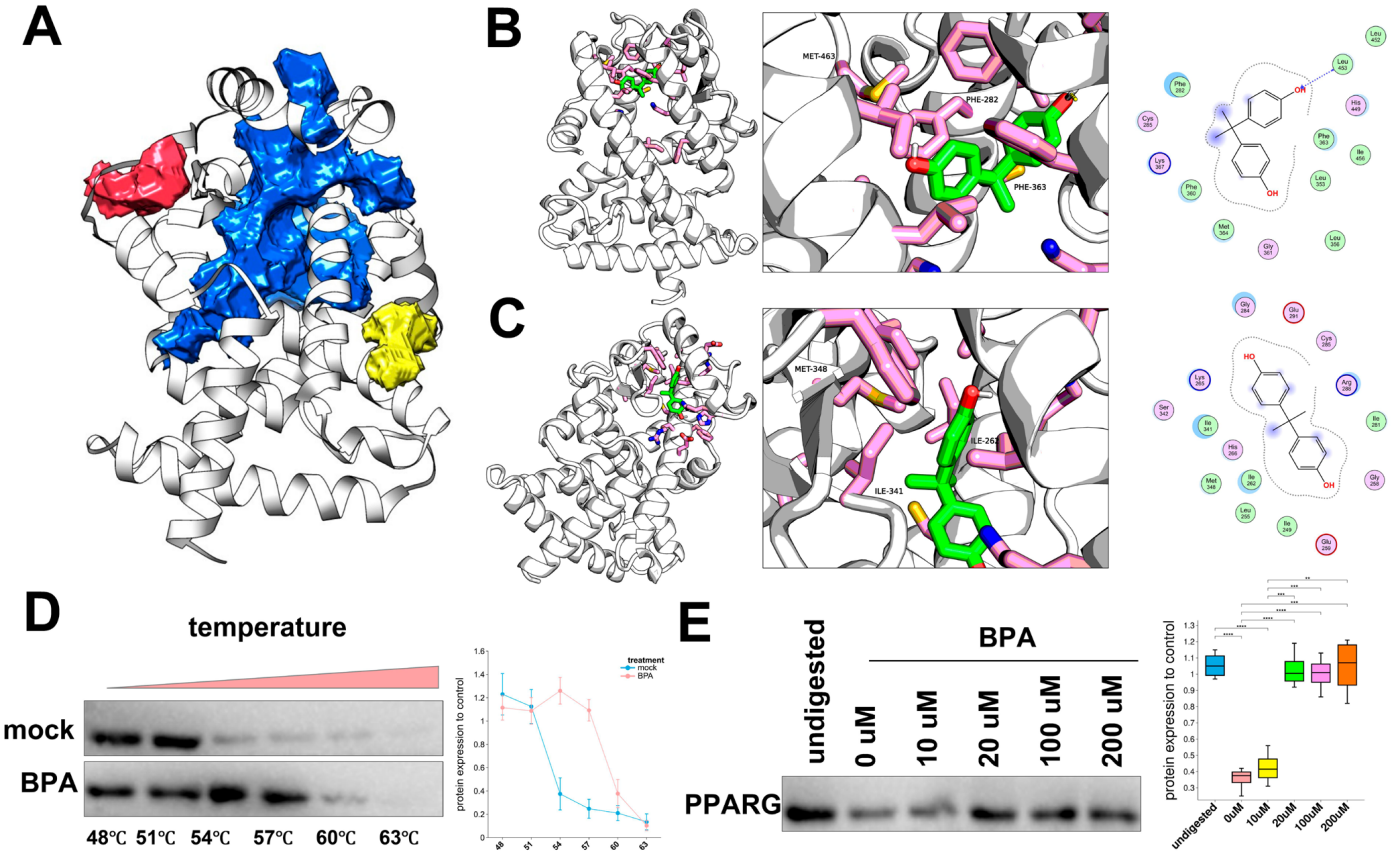
Structural analysis of BPA binding to PPARG. (A) Three‐dimensional structure of PPARG highlighting the three main active pockets, with the largest active pocket in blue. (B) Docking simulation of BPA within the largest active pocket of PPARG, showing BPA's interaction with surrounding residues. (C) Docking simulation of BPA within the second active pocket of PPARG, illustrating hydrogen bonds and hydrophobic interactions. (D) Cellular thermal shift assay (CESTA) showing the thermal stability of PPARG in the presence of BPA, with a temperature gradient from 48°C to 63°C. (E) Drug affinity responsive target stability (DARTS) analysis of PPARG protein levels in AML12 cells treated with increasing concentrations of BPA (0–200 μM), with quantification of protein expression relative to control. (Statistical significance: *p < 0.05, **p < 0.01, ***p < 0.001.)

Computational analysis revealed three major binding pockets within PPARG, with the largest (volume: 2671.46 Å^3^, surface area: 2676.31 Å^2^) defined by helices H6, H8, and H10 (Figure 5A). Molecular docking studies positioned BPA optimally within this pocket, exhibiting partial solvent exposure and forming a hydrogen bond with Leu453. Its mobility was restricted by surrounding hydrophobic residues (Figure 5B). A secondary binding site also showed partial solvent exposure, constrained by both hydrophobic and polar residues (Figure 5C). MM‐PBSA calculations yielded a binding energy of −83.67 ± 14.21 kcal/mol, suggesting a stable BPA‐PPARG interaction. Van der Waals forces were the primary contributors, with electrostatic and desolvation forces also contributing. Analysis of MM‐PBSA energy components (5–20 ns simulation) and per‐residue decomposition implicated residues 80 and 120–160 as key interaction sites (Figure S3A–G).

Cellular thermal shift assay (CETSA) confirmed increased PPARG thermal stability upon BPA binding (Figure 5D). Furthermore, drug affinity responsive target stability (DARTS) assays, employing pronase digestion, demonstrated enhanced PPARG stability in the presence of 50, 100, and 200 μM BPA compared to DMSO controls (Figure 5E). These findings strongly suggest a direct interaction between BPA and PPARG, leading to increased protein stability.

### 3.6 Molecular Dynamics Simulations Reveal BPA's Effects on PPARG Structure and Interaction

**FIGURE 6:**
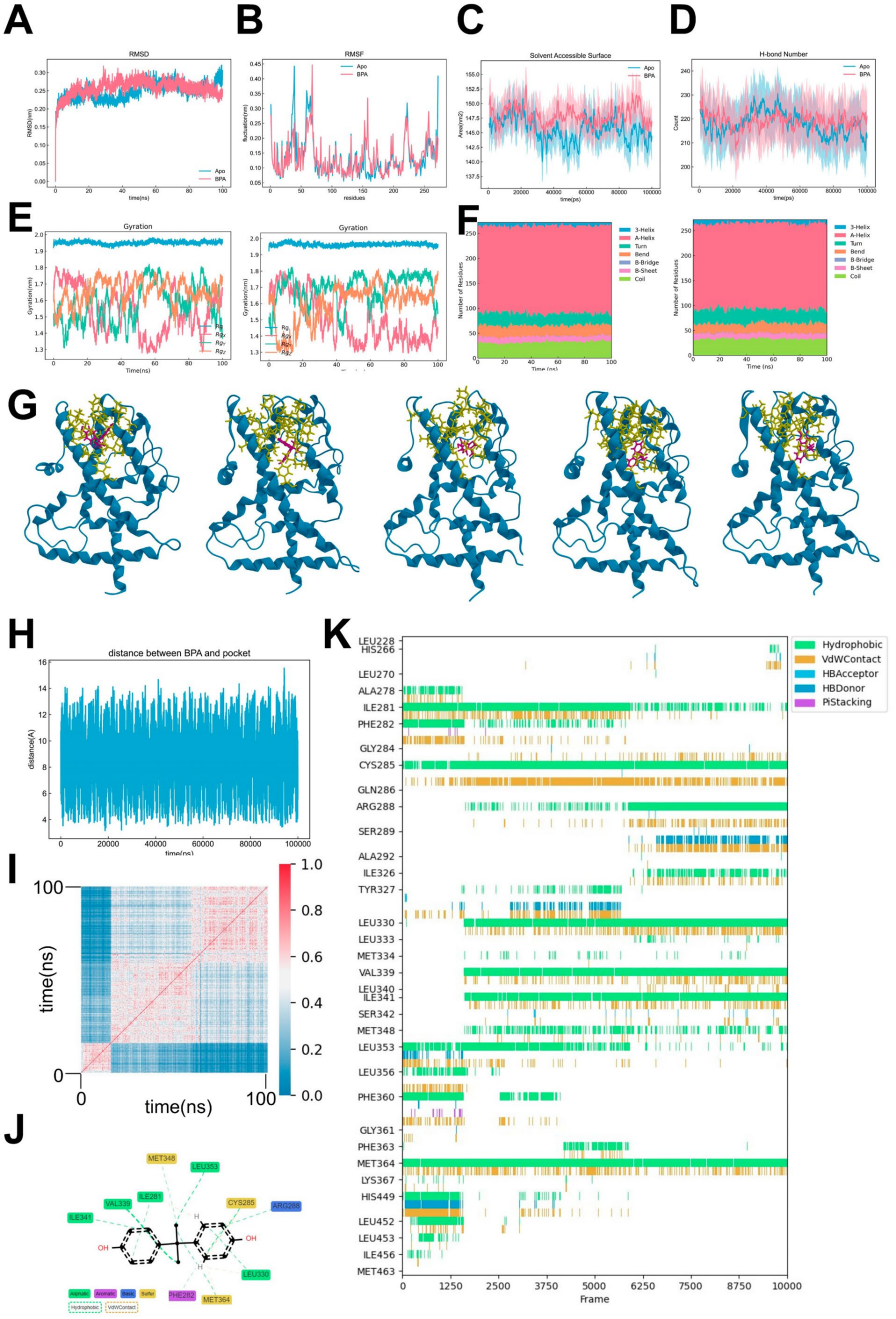
Molecular dynamics simulation of BPA binding to PPARG (A) Root mean square deviation (RMSD) of the backbone atoms for PPARG‐Apo (blue) and PPARG‐BPA (red) over 100 ns simulation time. (B) Root mean square fluctuation (RMSF) of the Cα atoms for PPARG‐Apo (blue) and PPARG‐BPA (red), indicating regions of flexibility. (C) Solvent accessible surface area (SASA) for PPARG‐Apo (blue) and PPARG‐BPA (red) during the simulation. (D) Number of hydrogen bonds in PPARG‐Apo (blue) and PPARG‐BPA (red) throughout the simulation. (E) Radius of gyration (Rg) for the xyz axes of PPARG‐Apo (left) and PPARG‐BPA (right), reflecting protein compactness. (F) Secondary structure content over time for PPARG‐Apo (left) and PPARG‐BPA (right) as analysed by DSSP. (G) Structural snapshots of BPA within the PPARG binding pocket at different time points (0, 25, 50, 75, and 100 ns). (H) Distance between BPA and the center of the PPARG binding pocket throughout the simulation. (I) Tanimoto similarity matrix for BPA assessing the similarity of BPA molecular fingerprints (bitvectors) over the course of the dynamics, reflecting conformational changes of BPA during the simulation. (J) Two‐dimensional interaction diagram of BPA within the PPARG binding pocket, highlighting key interactions. (K) Interaction timeline showing the persistence of various interactions between BPA and PPARG residues over the simulation period.

To elucidate the interaction between BPA and PPARG, we conducted a 100 ns molecular dynamics simulation on both unliganded PPARG (PPARG‐Apo) and BPA‐bound PPARG (PPARG‐BPA). The root mean square deviation (RMSD) for both systems stabilised rapidly, while the root mean square fluctuation (RMSF) exhibited consistent patterns, with notable decreases in fluctuations within the H2‐H3 and H3‐H4 linkers of PPARG‐BPA (Figure 6A,B). The solvent‐accessible surface area (SASA) and internal hydrogen bonds in PPARG‐Apo remained stable, and the radius of gyration indicated consistent compactness for both systems (Figure 6C,E).

DSSP analysis revealed similar secondary structure compositions for PPARG‐Apo and PPARG‐BPA (Figure 6F). BPA's dynamics within the active pocket were illustrated at various time points, showing periodic movement (Figure 6G). The Tanimoto similarity matrix assessed BPA's molecular fingerprints, reflecting conformational changes during the simulation (Figure 6I). The active pocket's morphology remained stable, indicating preservation throughout the dynamics.

Interactions between PPARG residues and BPA were primarily hydrophobic, with VAL339 being the most significant contributor. Dynamic interaction analysis showed SER289 and TYR327 acting as hydrogen bond donors at different simulation stages, while CYS285 and MET364 maintained consistent hydrophobic interactions with BPA (Figure 6J,K).

In dihedral angle‐based PCA analysis, PPARG‐Apo formed two conformational clusters corresponding to early and late simulation stages, while PPARG‐BPA exhibited multiple discontinuous clusters, indicating allosteric changes (Figure S4A). Dimensionality reduction techniques uniform manifold approximation and projection (UMAP), t‐distributed stochastic neighbour embedding (t‐SNE), and time‐structure independent component analysis (tICA) revealed a continuous flow pattern, suggesting ongoing conformational changes (Figure S4B–D).

Free energy landscape (FEL) analysis showed PPARG‐Apo with two energy traps, while PPARG‐BPA exhibited multiple irregular traps, with the lowest energy conformation appearing later in the simulation, indicating conformational evolution (Figure S5A,B). Inter‐residue distance analysis demonstrated consistent patterns for both systems, with PPARG‐BPA showing changes in correlation coefficients over time. Specifically, the distance between the N‐terminus of H10 and other residues decreased, while the distance between the C‐terminus of H13 and residues H1 to H8 increased (Figure S6A–E). PCA analysis based on residue distance correlation matrices (RDCM) indicated that neither PPARG‐Apo nor PPARG‐BPA formed independent conformational ensembles, suggesting minimal allosteric changes (Figure S6F).

Network analysis revealed that the two‐dimensional topological network of PPARG‐BPA was more divergent and radial compared to PPARG‐Apo, particularly in the connections between H7‐H11 and H11‐H12 (Figure S7A,B). Dynamic cross‐correlation matrix (DCCM) analysis indicated weakened inter‐residue correlations in PPARG‐BPA, including reduced cooperative correlations among adjacent residues along the principal axis (Figure S7C–F). Notably, antagonistic movements between secondary structures, such as H3 and H5, H3 and H10, and H3 and H13, diminished. Given that PPARG's agonistic activity relies on coactivator binding, these changes suggest a potential antagonistic effect of BPA on PPARG, highlighting the need for further investigation into BPA's impact on PPARG‐mediated lipid metabolism.

### 3.7 Structural and Functional Analysis of PPARG Homologues Across Species and Their Interaction With BPA

**FIGURE 7:**
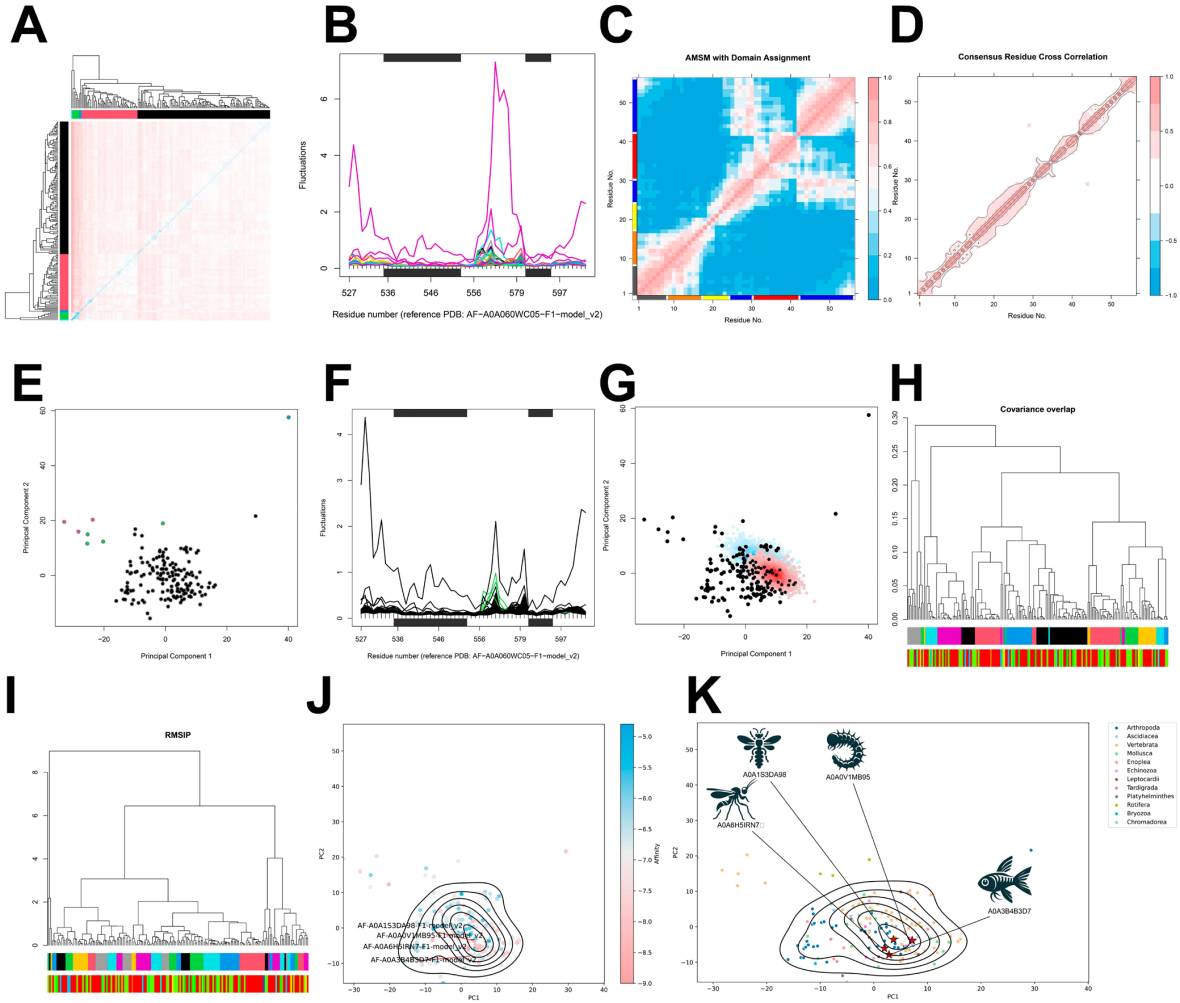
Structural similarity and conservation analysis of PPARG homologues. (A) RMSIP similarity matrix showing high structural similarity among PPARG homologues across different species. (B) Residue fluctuation peak plots indicating two distinct peaks within conserved segments, excluding N‐ and C‐terminal regions. (C) AMSM analysis with domain assignment revealing conserved residue segments, particularly in major helices and the H2‐H3 linker region. (D) Consensus residue cross‐correlation matrix demonstrating predominant correlation between neighbouring residues along the main structural axis. (E) Projection onto principal component axes dividing the structures into four clusters, highlighting significant residue fluctuation differences. (F) RMSD‐based fluctuation analysis showing differences between clusters 1 and 2, particularly in peak 1. (G) Principal component projection of dynamics‐derived conformations (PPARG‐Apo and PPARG‐BPA), showing overlap with homologous structures from certain species. (H) Hierarchical clustering based on covariance overlap compared with species‐based classifications, indicating no clear correspondence with species classifications. (I) Hierarchical clustering based on RMSIP showing local branches with consistency between structural feature‐based clustering and species classification. (J) PCA‐based projection illustrating overlap of dynamics‐derived conformations with homologous structures from various species. (K) Classification annotations for different species, displaying the corresponding structures of those with the highest affinities.

We utilised Foldseek to search for homologous proteins across different species based on structural similarity to human PPARG. Following the removal of redundant structures based on root mean square deviation (RMSD), subsequent analyses were conducted. The similarity matrix derived from RMSIP indicated that most structures maintained a high degree of similarity (Figure 7A). The residue fluctuation peak analysis revealed two prominent peaks (peak 1 and peak 2) within the conserved residue segments, aside from the N‐ and C‐termini (Figure 7B).

Using AMSM, we identified the major domains within these conserved residue segments, which included four primary helices and the connecting region between H2 and H3, excluding the N‐ and C‐terminal portions (Figure 7C). Consistency in the DCCM analysis of the conserved residue segments demonstrated significant cooperative correlations among adjacent residues along the principal axis of the structure (Figure 7D). The projection onto the principal component (PC) axes divided these structures into four clusters, with the differences in residue fluctuations between cluster 1 and cluster 3 primarily reflected in the previously identified peak 1 (Figure 7E,F).

We projected the conformations obtained from dynamics (PPARG‐Apo and PPARG‐BPA) into this space, revealing that homologous structures of PPARG from various species could overlap with the conformations sampled during dynamics, suggesting a degree of synchrony in structural evolution across different time scales (Figure 7G). Furthermore, we performed hierarchical clustering based on covariance overlap and RMSIP, comparing the classification results with the corresponding species taxonomy. Notably, no clear correspondence between structural classification and species taxonomy was observed in the major branches, although some consistency was noted in local branches (Figure 7H,I).

We conducted batch molecular docking of the homologous structures of PPARG from different species with BPA, calculating their affinities. Based on species annotations, the highest affinities for BPA were observed in the probable nuclear hormone receptor HR3 from the *Asian citrus psyllid*, the putative nuclear hormone receptor HR3 from *Trichinella papuae*, the nuclear hormone receptor HR3 from *Trichogramma brassicae*, and the thyroid hormone receptor alpha b from *Periophthalmus magnuspinnatus* (Figure 7J,K).

## 4 Discussion

The exposure to BPA has emerged as a significant concern due to its profound effects on lipid metabolism, particularly within the liver, which serves as the primary target organ for BPA toxicity [17, 18, 19, 20, 21]. Our findings indicate that low‐dose BPA exposure results in a more extensive impact on hepatic transcriptomic profiles compared to high‐dose exposure. Notably, we observed a significant downregulation of the key β‐oxidation gene, Cpt1a, alongside a marked suppression of PPARG transcriptional activity inferred from various analytical algorithms [13, 14]. This suggests that even low levels of BPA can disrupt normal hepatic functions, leading to metabolic disturbances.

In both in vivo and in vitro studies, we validated the perturbations in hepatic lipid metabolism induced by varying doses of BPA. These disturbances manifested as abnormal serum lipid levels and increased lipid accumulation within the liver. Mechanistically, we established that the downregulation of Cpt1a is a downstream effect of the inhibited transcriptional activity of PPARG due to BPA exposure. This reduction in Cpt1a abundance compromises the liver's β‐oxidation capacity, exacerbating lipid metabolic dysregulation [13, 14]. The implications of these findings are significant, as they highlight the potential for low‐dose BPA exposure to induce metabolic disorders through alterations in key regulatory pathways. Notably, at lower BPA concentrations, heightened sensitivity of certain nuclear regulators may lead to a pronounced suppression of Cpt1a, whereas higher doses can invoke compensatory mechanisms that override this inhibitory effect. Furthermore, the marked upregulation of Cpt1a upon PPARG overexpression underscores the direct regulatory relationship between PPARG and Cpt1a, revealing a nuanced interplay between BPA dosage, PPARG activity, and hepatic lipid metabolism.

Further investigation into the molecular interactions between BPA and PPARG revealed critical insights. Molecular docking and MM‐PBSA analyses indicated a direct interaction between BPA and PPARG, which was subsequently confirmed through cellular thermal shift assays (CETSA) and drug affinity responsive target stability (DARTS) experiments. These studies elucidated the binding dynamics and stability of PPARG in the presence of BPA, suggesting that BPA binding alters the structural integrity and functional capacity of PPARG [22, 23].

The molecular dynamics simulations provided a novel perspective on the dynamic interactions between BPA and PPARG. Our analysis revealed a complex network of hydrophobic interactions, van der Waals contacts, and non‐continuous hydrogen bonding that govern the behaviour of BPA within the PPARG binding pocket. The periodic movement of BPA within this pocket led to heterogeneous conformational changes in PPARG, characterised by multiple conformational switches compared to the apo state. This conformational flexibility may underlie the observed reduction in inter‐residue correlations, which is critical for the transcriptional activation of PPARG, as it relies heavily on the formation of protein–protein interaction interfaces with coactivators.

BPA exposure disrupts PPARG transcriptional activity by interfering with its binding to response elements on target genes, including CPT1A. This interference diminishes β‐oxidation by downregulating CPT1A expression, thereby promoting hepatic lipid accumulation. Our findings demonstrate a dose‐dependent mechanism, where lower BPA concentrations intensify the sensitivity of nuclear regulators and co‐repressors involved in PPARG signalling, whereas higher doses activate compensatory pathways that partially counteract these effects. Collectively, these results underscore the pivotal role of the PPARG–CPT1A axis in maintaining hepatic lipid homeostasis and reveal how BPA‐induced disruptions in this regulatory network exacerbate metabolic imbalances.

Moreover, our cross‐species analysis of PPARG homologues based on structural features unveiled evolutionary characteristics that may inform our understanding of BPA's effects across different organisms [22, 24, 25, 26]. We identified several proteins with high affinity for BPA, suggesting potential implications for wildlife and ecosystem health, as these interactions may disrupt metabolic processes in non‐target species.

This study reveals that BPA exerts detrimental effects on hepatic lipid metabolism by directly disrupting the PPARG–CPT1A axis, a crucial pathway for β‐oxidation and lipid homeostasis. By downregulating PPARG transcriptional activity, BPA diminishes CPT1A expression, thereby compromising fatty acid oxidation and exacerbating lipid accumulation in the liver. Cross‐species analyses demonstrate that BPA similarly impairs this regulatory axis in multiple animal models, highlighting a potentially widespread threat to metabolic health. Our findings provide novel insights into the mechanistic underpinnings of BPA‐induced metabolic disturbances and underscore the urgency of further research on BPA's biological impact, particularly within the framework of regulatory policies and public health. Future investigations should focus on the long‐term consequences of BPA exposure for liver health and metabolic disorders, including potential epigenetic modifications in hepatic cells [27, 28, 29]. In addition, examining BPA's interactions with other metabolic pathways and nuclear receptors will be essential for a comprehensive understanding of its toxicological profile and the development of effective mitigation strategies [30].

## Author Contributions

**Xiliang Zhu:** conceptualization (equal), data curation (equal), funding acquisition (equal), methodology (equal), validation (equal), visualization (equal), writing – original draft (equal). **Qi Liu:** conceptualization (equal), data curation (equal), resources (equal), software (equal), validation (equal). **Zhaoyun Cheng:** conceptualization (equal), data curation (equal), software (equal), supervision (equal), writing – original draft (equal). **Yi Luo:** data curation (equal), investigation (equal), methodology (equal), visualization (equal), writing – original draft (equal), writing – review and editing (equal).

## Consent

The authors have nothing to report.

## Conflicts of Interest

The authors declare no conflicts of interest.

## Supporting information


**Figure S1.** Effects of different doses of BPA on liver function‐related parameters in mice. The figure illustrates the levels of albumin (ALB), alkaline phosphatase (ALP), alanine aminotransferase (ALT), aspartate aminotransferase (AST), and total protein (TP). VEH represents the control group, while LOW and HI denote the low and high dose BPA treatment groups, respectively. The results indicate that varying doses of BPA did not significantly affect these liver function parameters.

**Figure S2.** BPA effects on hepatic mitochondrial membrane potential and reactive oxygen species. (A) TMRE. Representative images of cells stained with TMRE (red) to evaluate mitochondrial membrane potential; nuclei are counterstained with DAPI (blue). The box plot shows relative fluorescence intensity for each treatment. (B) DCFH‐DA. Flow cytometry histograms illustrating intracellular ROS levels detected by DCFH‐DA, with the box plot summarising relative fluorescence intensity across treatment groups.

**Figure S3.** MM‐PBSA energy calculations for PPARG‐BPA system (A) Average MM‐PBSA energy components, showing contributions from van der Waals (vdW), coulombic (cou), molecular mechanics (MM), polar solvation (dPB), and non‐polar solvation (dSA) energies. (B) Molecular mechanics (MM) energy components (vdW, cou, and MM) over time during the simulation. (C) Solvent accessible surface area (SA) energy components for the complex, ligand, and protein over time. (D) Polar solvation (PB) energy components for the complex, ligand, and protein over time. (E) Residue‐wise average molecular mechanics energy (MM = cou + vdW) contributions. (F) Residue‐wise average polar solvation energy (PB) contributions. (G) Residue‐wise average solvent accessible surface area (SA) energy contributions.

**Figure S4.** Conformational clustering and dimensionality reduction analysis of PPARG‐Apo and PPARG‐BPA (A) Principal component analysis (PCA) based on dihedral angles for PPARG‐Apo (left) and PPARG‐BPA (right), showing conformational clusters over time. (B) Uniform manifold approximation and projection (UMAP) based on atomic coordinates for PPARG‐Apo (left) and PPARG‐BPA (right), illustrating continuous conformational evolution. (C) t‐distributed stochastic neighbour embedding (t‐SNE) analysis on atomic coordinates for PPARG‐Apo (left) and PPARG‐BPA (right), indicating conformational transitions over time. (D) Time‐lagged independent component analysis (tICA) on atomic coordinates for PPARG‐Apo (left) and PPARG‐BPA (right), showing distinct conformational clusters.

**Figure S5.** Free energy landscape (FEL) analysis of PPARG conformations (A) Left: Gibbs energy landscape of PPARG‐Apo showing two irregular energy basins. The colour scale represents the Gibbs free energy (G) in kJ/mol, with darker blue indicating lower energy states. Right: scatter plot of the same data, with colour indicating simulation time. Minimum energy states are observed at 49.9 ns, 52.41 ns, and 50.38 ns. (B) Left: Gibbs energy landscape of PPARG‐BPA revealing three irregular energy basins. The colour scale is consistent with panel A. Right: corresponding scatter plot with colour indicating simulation time. Minimum energy states are identified at 38.4 ns, 67.49 ns, 35.39 ns, 76.37 ns, and 82.4 ns.

**Figure S6.** Inter‐residue distance and correlation analysis of PPARG‐Apo and PPARG‐BPA (A) average distance matrix of inter‐residue distances for PPARG‐Apo (left) and PPARG‐BPA (right). Colour scale indicates distance in nm. (B) Time occupancy of the contact matrix for PPARG‐Apo (left) and PPARG‐BPA (right). Colour scale represents occupancy from 0 to 1. (C) Averaged formation time of encounters between residues for PPARG‐Apo (left) and PPARG‐BPA (right). Colour scale indicates time in arbitrary units. (D) Dynamic cross‐correlation matrix (DCCM) of the residue distance correlation matrix (RDCM) for PPARG‐Apo (left) and PPARG‐BPA (right). Colour scale ranges from −1 (anticorrelated) to 1 (correlated). (E) Pearson correlation matrix over time for PPARG‐Apo (left) and PPARG‐BPA (right). Colour scale ranges from −1 (negative correlation) to 1 (positive correlation). (F) Principal component analysis (PCA) of the RDCM for PPARG‐Apo (left) and PPARG‐BPA (right). Points are coloured by simulation time, with blue representing early timepoints and red representing later timepoints.

**Figure S7.** Structural network and residue cross‐correlation analysis of PPARG‐Apo and PPARG‐BPA (A) shortest path network representation of PPARG structures with coloured nodes indicating key residues; left panels show PPARG‐Apo and right panels show PPARG‐BPA. (B) Second shortest path network of PPARG structures, with colour‐coded residues, illustrating significant pathways; left panels depict PPARG‐Apo and right panels illustrate PPARG‐BPA. (C) Residue cross‐correlation matrix for PPARG‐Apo, displaying correlations between residues with red indicating positive correlation and blue indicating negative correlation. (D) Residue cross‐correlation matrix for PPARG‐BPA, highlighting altered correlations due to BPA binding. (E) 3D representation of residue correlation in PPARG‐Apo, visualised from different angles to demonstrate connectivity. (F) 3D visualisation of residue correlation in PPARG‐BPA, shown from various perspectives to illustrate changes induced by BPA binding.

## Data Availability

Data available in article Supporting Information.

## References

[1] R. Emfietzoglou , N. Spyrou , C. S. Mantzoros , and M. Dalamaga , “Could the Endocrine Disruptor Bisphenol‐A Be Implicated in the Pathogenesis of Oral and Oropharyngeal Cancer? Metabolic Considerations and Future Directions,” Metabolism 91 (2019): 61–69, 10.1016/j.metabol.2018.11.007. 30458176

[2] X. Hong , Y. Zhou , Z. Zhu , et al., “Environmental Endocrine Disruptor Bisphenol A Induces Metabolic Derailment and Obesity via Upregulating IL‐17a in Adipocytes,” Environment International 172 (2023): 107759, 10.1016/j.envint.2023.107759. 36696794

[3] Y. Ma , H. Liu , J. Wu , et al., “The Adverse Health Effects of Bisphenol A and Related Toxicity Mechanisms,” Environmental Research 176 (2019): 108575, 10.1016/j.envres.2019.108575. 31299621

[4] V. Araiza , M. S. Mendoza , K. Castro , et al., “Bisphenol A, an Endocrine‐Disruptor Compund, That Modulates the Immune Response to Infections,” Frontiers in Bioscience‐Landmark 26, no. 2 (2021): 346–362, 10.2741/4897. 33049673

[5] A. Abraham and P. Chakraborty , “A Review on Sources and Health Impacts of Bisphenol a,” Reviews on Environmental Health 35, no. 2 (2020): 201–210, 10.1515/reveh-2019-0034. 31743105

[6] B. Attema , O. Kummu , S. Pitkanen , et al., “Metabolic Effects of Nuclear Receptor Activation In Vivo After 28‐Day Oral Exposure to Three Endocrine‐Disrupting Chemicals,” Archives of Toxicology 98, no. 3 (2024): 911–928, 10.1007/s00204-023-03658-2. 38182912 PMC10861694

[7] A. Kodila , N. Franko , and D. M. Sollner , “A Review on Immunomodulatory Effects of BPA Analogues,” Archives of Toxicology 97, no. 7 (2023): 1831–1846, 10.1007/s00204-023-03519-y. 37204436 PMC10256647

[8] M. J. Kim and Y. J. Park , “Bisphenols and Thyroid Hormone,” Endocrinology and Metabolism 34, no. 4 (2019): 340–348, 10.3803/EnM.2019.34.4.340. 31884733 PMC6935774

[9] V. Rouiller‐Fabre , M. J. Guerquin , T. N'Tumba‐Byn , et al., “Nuclear Receptors and Endocrine Disruptors in Fetal and Neonatal Testes: A Gapped Landscape,” Frontiers in Endocrinology 6 (2015): 58, 10.3389/fendo.2015.00058. 25999913 PMC4423451

[10] L. Shi , L. J. Li , X. Y. Sun , et al., “Er‐Dong‐Xiao‐Ke Decoction Regulates Lipid Metabolism via PPARG‐Mediated UCP2/AMPK Signaling to Alleviate Diabetic Meibomian Gland Dysfunction,” Journal of Ethnopharmacology 333 (2024): 118484, 10.1016/j.jep.2024.118484. 38925318

[11] J. Boeckmans , A. Gatzios , A. Heymans , et al., “Transcriptomics Reveals Discordant Lipid Metabolism Effects Between In Vitro Models Exposed to Elafibranor and Liver Samples of NAFLD Patients After Bariatric Surgery,” Cells 11, no. 5 (2022): 893, 10.3390/cells11050893. 35269515 PMC8909190

[12] A. F. Silva , G. A. Abruzzese , M. J. Ferrer , et al., “Fetal Programming by Androgen Excess Impairs Liver Lipid Content and PPARg Expression in Adult Rats,” Journal of Developmental Origins of Health and Disease 13, no. 3 (2022): 300–309, 10.1017/S2040174421000416. 34275515

[13] Z. Liu , W. Liu , W. Wang , et al., “CPT1a‐Mediated Fatty Acid Oxidation Confers Cancer Cell Resistance to Immune‐Mediated Cytolytic Killing,” Proceedings of the National Academy of Sciences of the United States of America 120, no. 39 (2023): e1992089176, 10.1073/pnas.2302878120. PMC10523454 37722058

[14] K. Liang , “Mitochondrial CPT1a: Insights Into Structure, Function, and Basis for Drug Development,” Frontiers in Pharmacology 14 (2023): 1160440, 10.3389/fphar.2023.1160440. 37033619 PMC10076611

[15] B. W. Wong , X. Wang , A. Zecchin , et al., “The Role of Fatty Acid Beta‐Oxidation in Lymphangiogenesis,” Nature 542, no. 7639 (2017): 49–54, 10.1038/nature21028. 28024299

[16] M. T. Nakamura , B. E. Yudell , and J. J. Loor , “Regulation of Energy Metabolism by Long‐Chain Fatty Acids,” Progress in Lipid Research 53 (2014): 124–144, 10.1016/j.plipres.2013.12.001. 24362249

[17] W. He , Z. Gao , S. Liu , et al., “G Protein‐Coupled Estrogen Receptor Activation by Bisphenol‐A Disrupts Lipid Metabolism and Induces Ferroptosis in the Liver,” Environmental Pollution 334 (2023): 122211, 10.1016/j.envpol.2023.122211. 37454720

[18] L. Le Corre , P. Besnard , and M. C. Chagnon , “BPA, an Energy Balance Disruptor,” Critical Reviews in Food Science and Nutrition 55, no. 6 (2015): 769–777, 10.1080/10408398.2012.678421. 24915348

[19] Y. Zhang , S. Han , T. Li , L. Zhu , and F. Wei , “Bisphenol a Induces Non‐Alcoholic Fatty Liver Disease by Promoting the O‐GlcNAcylation of NLRP3,” Archives of Physiology and Biochemistry 130, no. 6 (2023): 1–9, 10.1080/13813455.2023.2288533. 38038745

[20] M. S. Rahman , W. K. Pang , S. Amjad , et al., “Hepatic Consequences of a Mixture of Endocrine‐Disrupting Chemicals in Male Mice,” Journal of Hazardous Materials 436 (2022): 129236, 10.1016/j.jhazmat.2022.129236. 35739755

[21] G. Sudhakaran , P. S. Priya , B. Haridevamuthu , et al., “Mechanistic Interplay of Dual Environmental Stressors: Bisphenol‐A and Cadmium‐Induced Ovarian Follicular Damage and Hepatocyte Dysfunction In Vivo,” Science of the Total Environment 924 (2024): 171706, 10.1016/j.scitotenv.2024.171706. 38490420

[22] H. Routti , R. Lille‐Langoy , M. K. Berg , et al., “Environmental Chemicals Modulate Polar Bear ( *Ursus maritimus* ) Peroxisome Proliferator‐Activated Receptor Gamma (PPARG) and Adipogenesis In Vitro,” Environmental Science & Technology 50, no. 19 (2016): 10708–10720, 10.1021/acs.est.6b03020. 27602593

[23] L. A. Hoepner , “Bisphenol A: A Narrative Review of Prenatal Exposure Effects on Adipogenesis and Childhood Obesity via Peroxisome Proliferator‐Activated Receptor Gamma,” Environmental Research 173 (2019): 54–68, 10.1016/j.envres.2019.03.012. 30897403 PMC10637253

[24] R. Yang , Y. Lu , N. Yin , and F. Faiola , “Transcriptomic Integration Analyses Uncover Common Bisphenol A Effects Across Species and Tissues Primarily Mediated by Disruption of JUN/FOS, EGFR, ER, PPARG, and P53 Pathways,” Environmental Science & Technology 57, no. 48 (2023): 19156–19168, 10.1021/acs.est.3c02016. 37978927

[25] L. Shu , Q. Meng , G. Diamante , et al., “Prenatal Bisphenol A Exposure in Mice Induces Multitissue Multiomics Disruptions Linking to Cardiometabolic Disorders,” Endocrinology 160, no. 2 (2019): 409–429, 10.1210/en.2018-00817. 30566610 PMC6349005

[26] A. Salehi , N. Loganathan , and D. D. Belsham , “Bisphenol A Induces Pomc Gene Expression Through Neuroinflammatory and PPARgamma Nuclear Receptor‐Mediated Mechanisms in POMC‐Expressing Hypothalamic Neuronal Models,” Molecular and Cellular Endocrinology 479 (2019): 12–19, 10.1016/j.mce.2018.08.009. 30149043

[27] M. K. Moon , I. K. Jeong , O. T. Jung , et al., “Long‐Term Oral Exposure to Bisphenol A Induces Glucose Intolerance and Insulin Resistance,” Journal of Endocrinology 226, no. 1 (2015): 35–42, 10.1530/JOE-14-0714. 25972359

[28] L. F. Azevedo , D. C. Porto , D. S. R. C. Cristina , C. M. Hornos , L. C. Alberici , and F. J. Barbosa , “Long‐Term Exposure to Bisphenol A or S Promotes Glucose Intolerance and Changes Hepatic Mitochondrial Metabolism in Male Wistar Rats,” Food and Chemical Toxicology 132 (2019): 110694, 10.1016/j.fct.2019.110694. 31344369

[29] W. Y. Chen , Y. P. Shen , and S. C. Chen , “Assessing Bisphenol A (BPA) Exposure Risk From Long‐Term Dietary Intakes in Taiwan,” Science of the Total Environment 543 (2016): 140–146, 10.1016/j.scitotenv.2015.11.029. 26580736

[30] V. Delfosse , M. Grimaldi , A. le Maire , W. Bourguet , and P. Balaguer , “Nuclear Receptor Profiling of Bisphenol‐A and Its Halogenated Analogues,” Vitamins and Hormones 94 (2014): 229–251, 10.1016/B978-0-12-800095-3.00009-2, New York Elsevier. 24388193

