# Supplementary figures and images for: Disruption of PPARG Activity and CPT1A Regulation by Bisphenol A: Implications for Hepatic Lipid Metabolism
Source: J Cell Mol Med. 2025 May 9;29(9):e70416. doi: 10.1111/jcmm.70416 (PMC12064412; doi:10.1111/jcmm.70416)

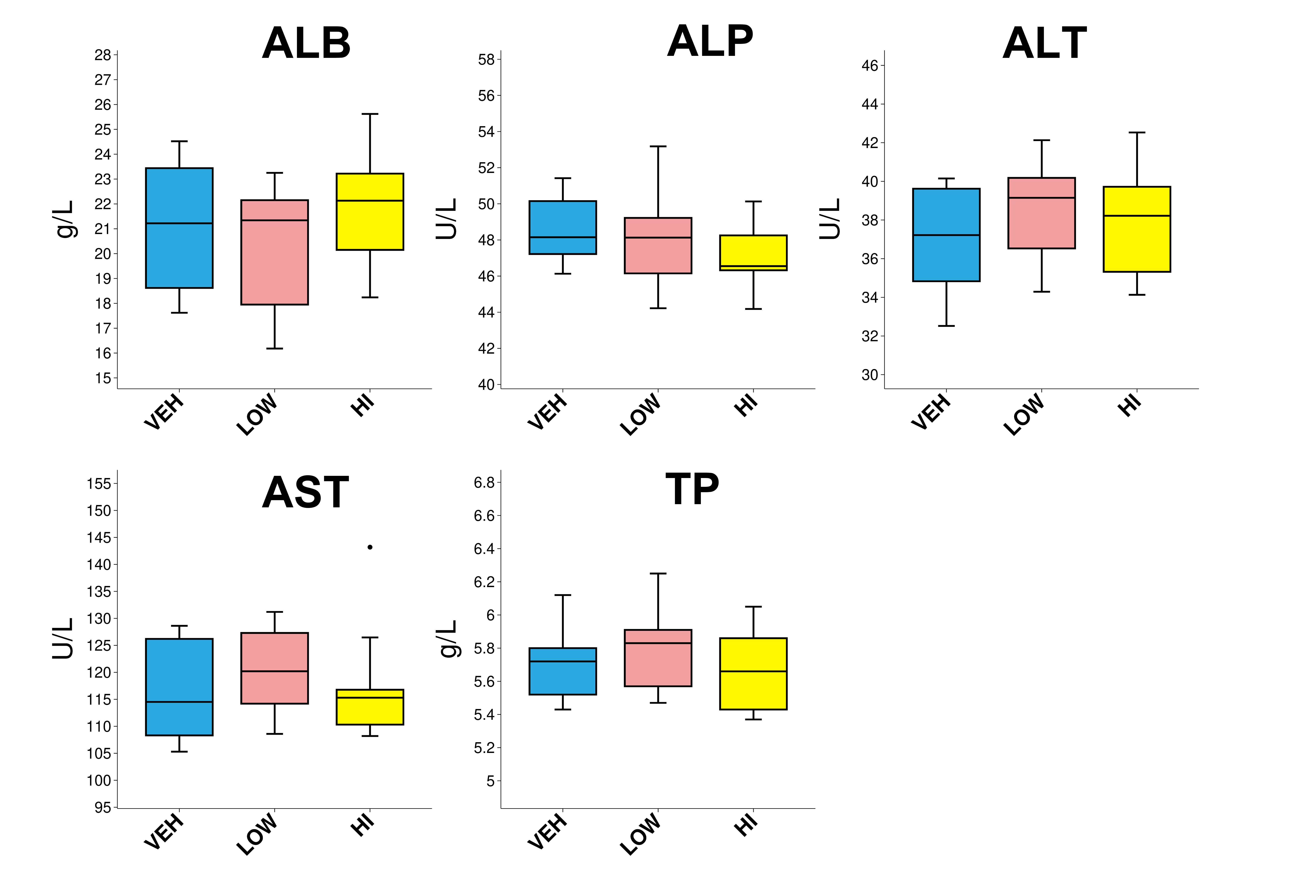

Supplement: Supplementary file 1 — Figure S1. Effects of different doses of BPA on liver function‐related parameters in mice. The figure illustrates the levels of albumin (ALB), alkaline phosphatase (ALP), alanine aminotransferase (ALT), aspartate aminotransferase (AST), and total protein (TP). VEH represents the control group, while LOW and HI denote the low and high dose BPA treatment groups, respectively. The results indicate that varying doses of BPA did not significantly affect these liver function parameters. [file JCMM-29-e70416-s007.jpg]

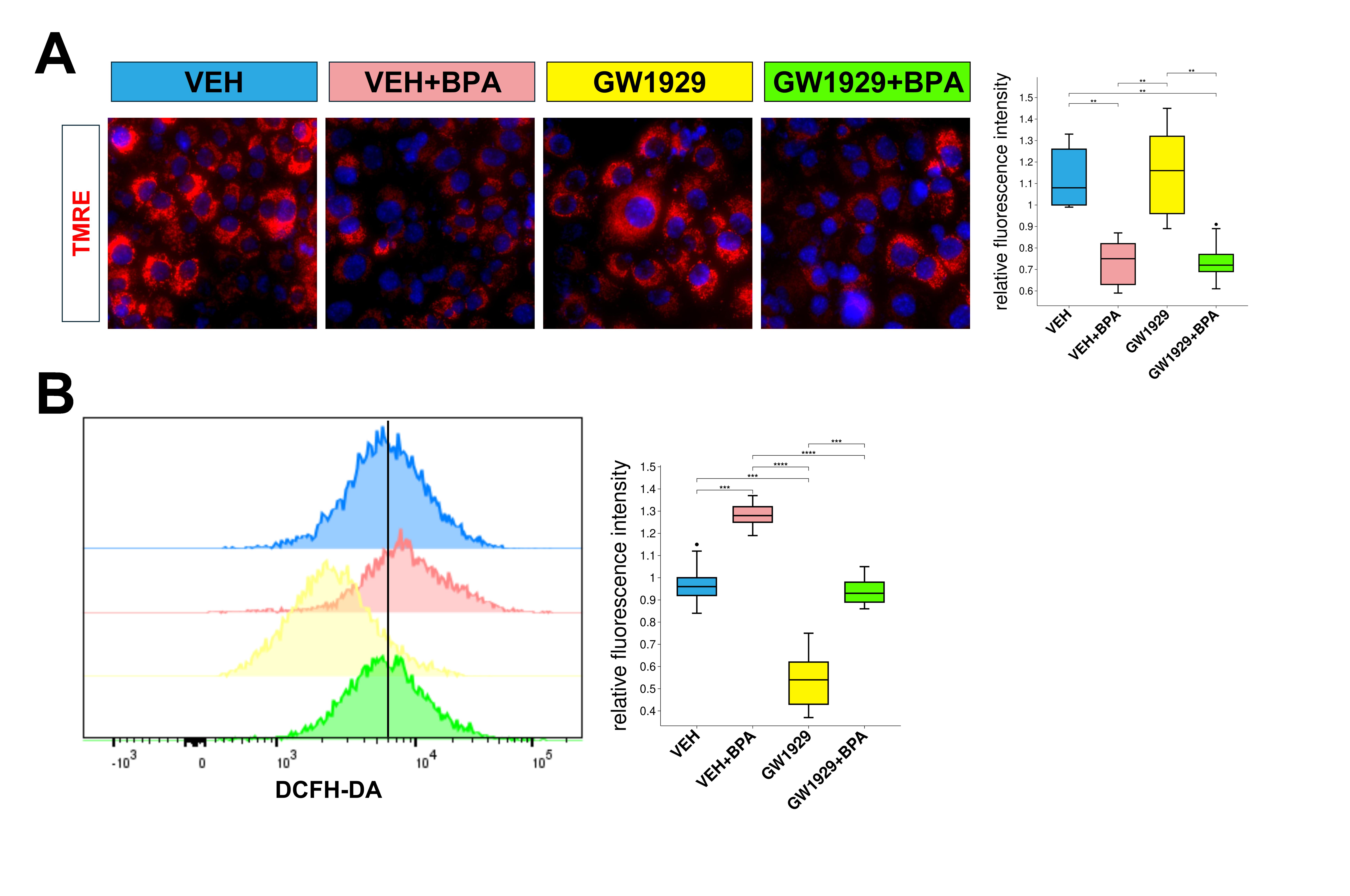

Supplement: Supplementary file 2 — Figure S2. BPA effects on hepatic mitochondrial membrane potential and reactive oxygen species. (A) TMRE. Representative images of cells stained with TMRE (red) to evaluate mitochondrial membrane potential; nuclei are counterstained with DAPI (blue). The box plot shows relative fluorescence intensity for each treatment. (B) DCFH‐DA. Flow cytometry histograms illustrating intracellular ROS levels detected by DCFH‐DA, with the box plot summarising relative fluorescence intensity across treatment groups. [file JCMM-29-e70416-s006.jpg]

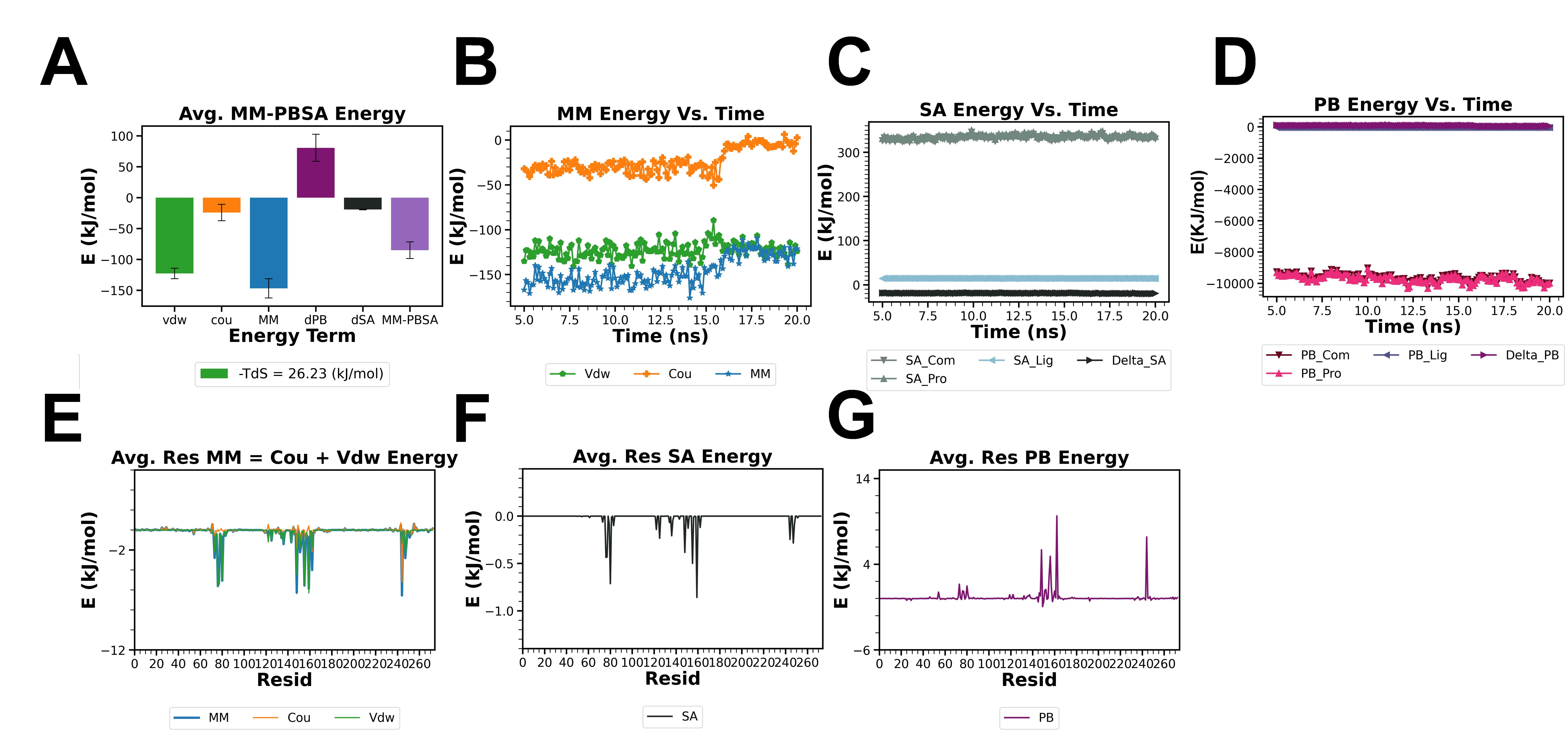

Supplement: Supplementary file 3 — Figure S3. MM‐PBSA energy calculations for PPARG‐BPA system (A) Average MM‐PBSA energy components, showing contributions from van der Waals (vdW), coulombic (cou), molecular mechanics (MM), polar solvation (dPB), and non‐polar solvation (dSA) energies. (B) Molecular mechanics (MM) energy components (vdW, cou, and MM) over time during the simulation. (C) Solvent accessible surface area (SA) energy components for the complex, ligand, and protein over time. (D) Polar solvation (PB) energy components for the complex, ligand, and protein over time. (E) Residue‐wise average molecular mechanics energy (MM = cou + vdW) contributions. (F) Residue‐wise average polar solvation energy (PB) contributions. (G) Residue‐wise average solvent accessible surface area (SA) energy contributions. [file JCMM-29-e70416-s002.jpg]

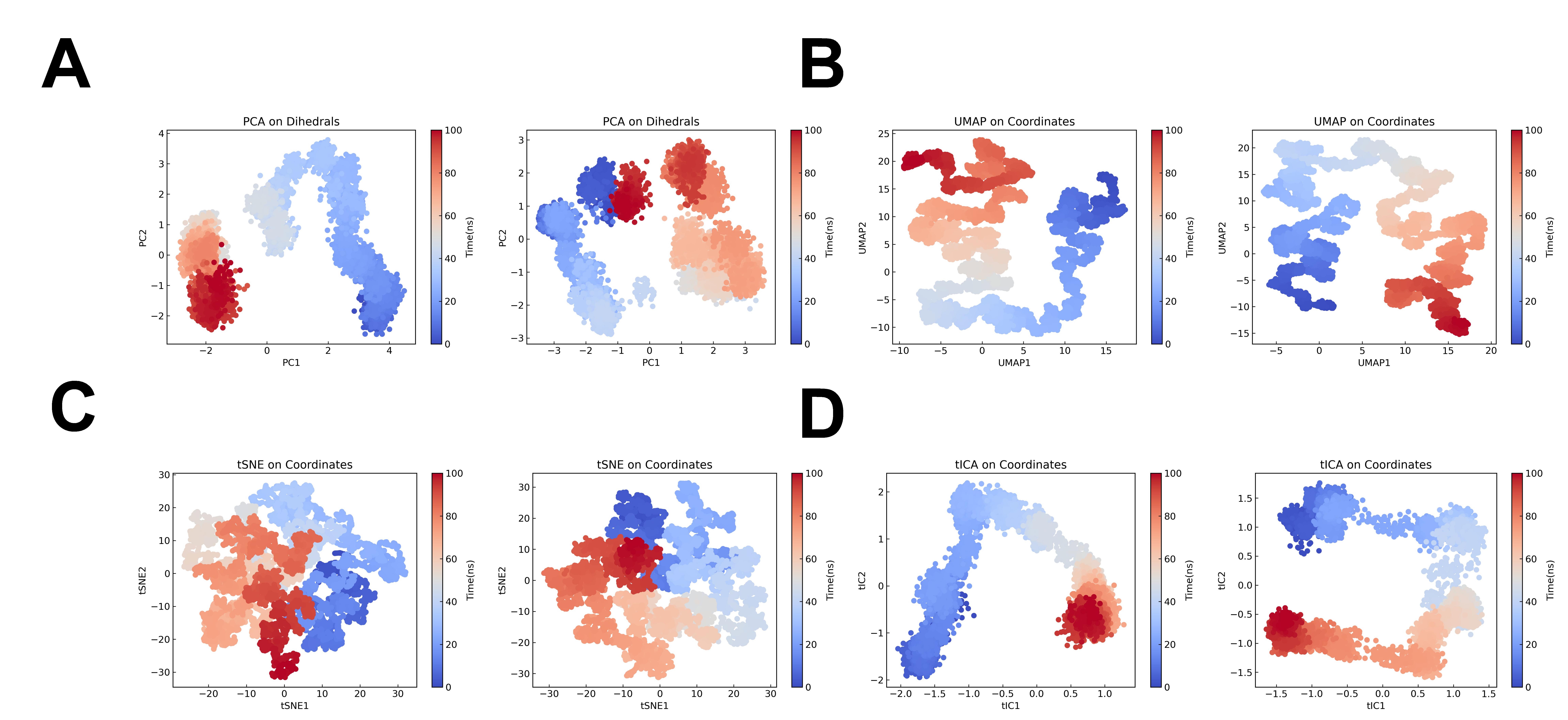

Supplement: Supplementary file 4 — Figure S4. Conformational clustering and dimensionality reduction analysis of PPARG‐Apo and PPARG‐BPA (A) Principal component analysis (PCA) based on dihedral angles for PPARG‐Apo (left) and PPARG‐BPA (right), showing conformational clusters over time. (B) Uniform manifold approximation and projection (UMAP) based on atomic coordinates for PPARG‐Apo (left) and PPARG‐BPA (right), illustrating continuous conformational evolution. (C) t‐distributed stochastic neighbour embedding (t‐SNE) analysis on atomic coordinates for PPARG‐Apo (left) and PPARG‐BPA (right), indicating conformational transitions over time. (D) Time‐lagged independent component analysis (tICA) on atomic coordinates for PPARG‐Apo (left) and PPARG‐BPA (right), showing distinct conformational clusters. [file JCMM-29-e70416-s003.jpg]

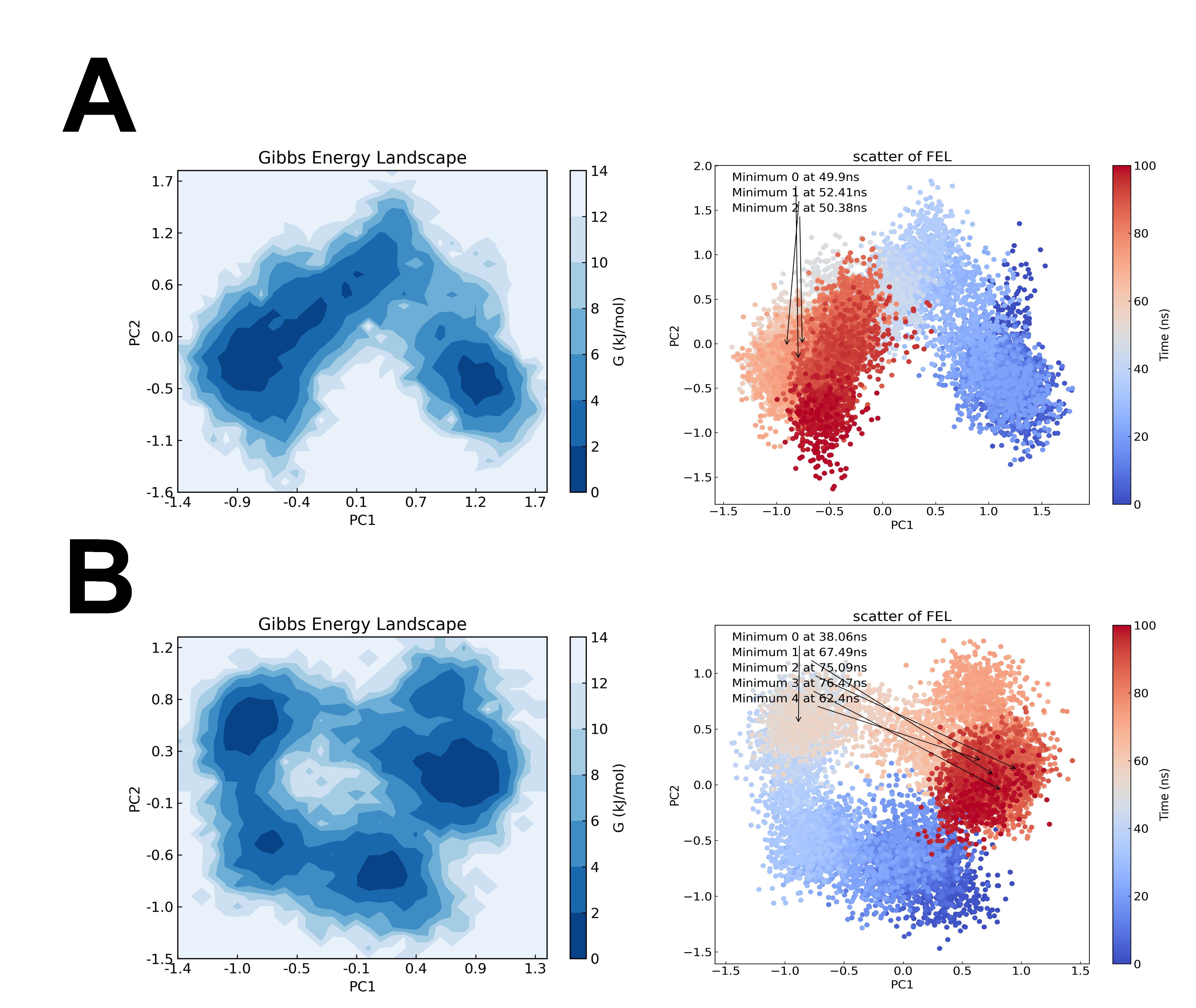

Supplement: Supplementary file 5 — Figure S5. Free energy landscape (FEL) analysis of PPARG conformations (A) Left: Gibbs energy landscape of PPARG‐Apo showing two irregular energy basins. The colour scale represents the Gibbs free energy (G) in kJ/mol, with darker blue indicating lower energy states. Right: scatter plot of the same data, with colour indicating simulation time. Minimum energy states are observed at 49.9 ns, 52.41 ns, and 50.38 ns. (B) Left: Gibbs energy landscape of PPARG‐BPA revealing three irregular energy basins. The colour scale is consistent with panel A. Right: corresponding scatter plot with colour indicating simulation time. Minimum energy states are identified at 38.4 ns, 67.49 ns, 35.39 ns, 76.37 ns, and 82.4 ns. [file JCMM-29-e70416-s001.jpg]

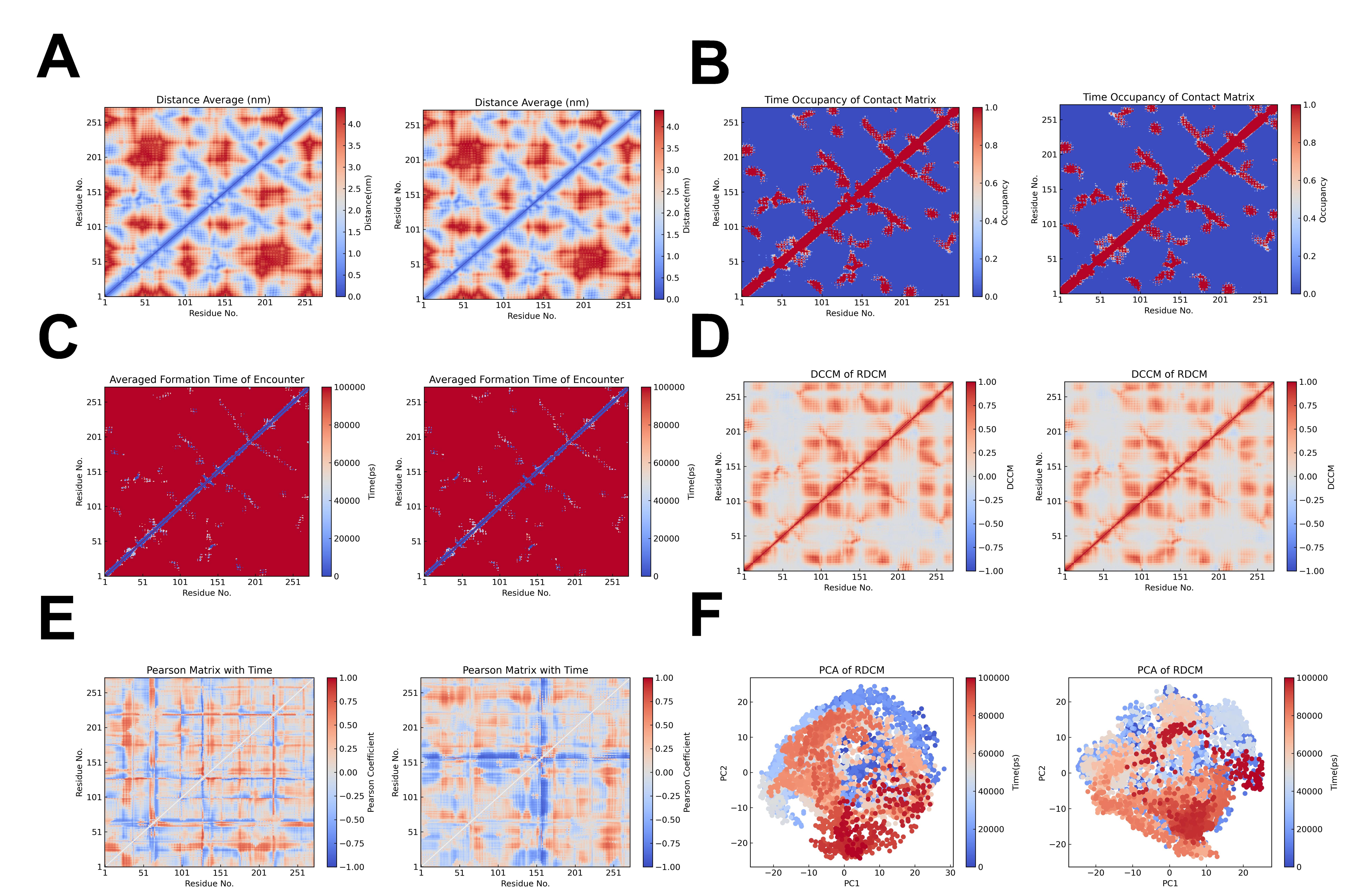

Supplement: Supplementary file 6 — Figure S6. Inter‐residue distance and correlation analysis of PPARG‐Apo and PPARG‐BPA (A) average distance matrix of inter‐residue distances for PPARG‐Apo (left) and PPARG‐BPA (right). Colour scale indicates distance in nm. (B) Time occupancy of the contact matrix for PPARG‐Apo (left) and PPARG‐BPA (right). Colour scale represents occupancy from 0 to 1. (C) Averaged formation time of encounters between residues for PPARG‐Apo (left) and PPARG‐BPA (right). Colour scale indicates time in arbitrary units. (D) Dynamic cross‐correlation matrix (DCCM) of the residue distance correlation matrix (RDCM) for PPARG‐Apo (left) and PPARG‐BPA (right). Colour scale ranges from −1 (anticorrelated) to 1 (correlated). (E) Pearson correlation matrix over time for PPARG‐Apo (left) and PPARG‐BPA (right). Colour scale ranges from −1 (negative correlation) to 1 (positive correlation). (F) Principal component analysis (PCA) of the RDCM for PPARG‐Apo (left) and PPARG‐BPA (right). Points are coloured by simulation time, with blue representing early timepoints and red representing later timepoints. [file JCMM-29-e70416-s005.jpg]

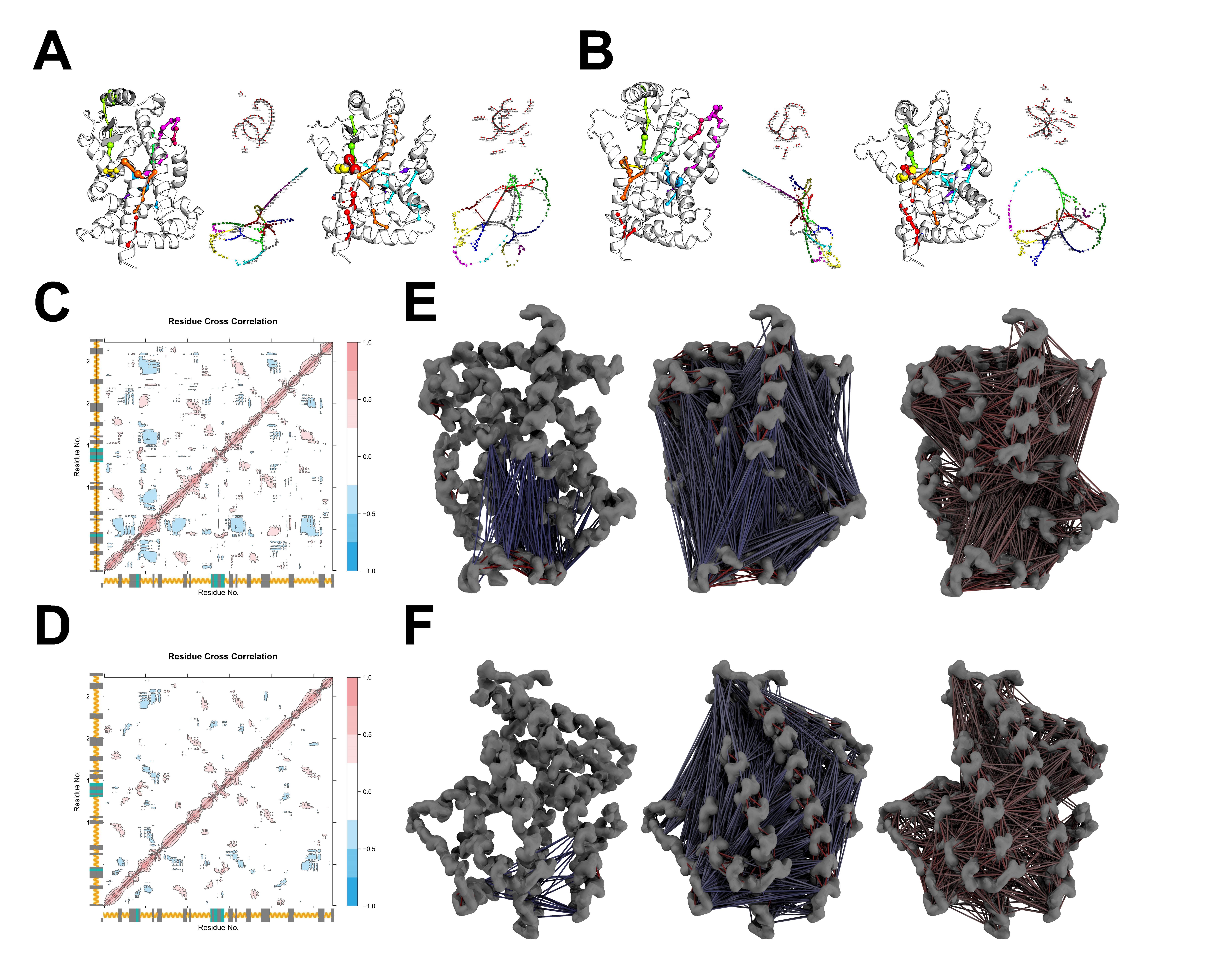

Supplement: Supplementary file 7 — Figure S7. Structural network and residue cross‐correlation analysis of PPARG‐Apo and PPARG‐BPA (A) shortest path network representation of PPARG structures with coloured nodes indicating key residues; left panels show PPARG‐Apo and right panels show PPARG‐BPA. (B) Second shortest path network of PPARG structures, with colour‐coded residues, illustrating significant pathways; left panels depict PPARG‐Apo and right panels illustrate PPARG‐BPA. (C) Residue cross‐correlation matrix for PPARG‐Apo, displaying correlations between residues with red indicating positive correlation and blue indicating negative correlation. (D) Residue cross‐correlation matrix for PPARG‐BPA, highlighting altered correlations due to BPA binding. (E) 3D representation of residue correlation in PPARG‐Apo, visualised from different angles to demonstrate connectivity. (F) 3D visualisation of residue correlation in PPARG‐BPA, shown from various perspectives to illustrate changes induced by BPA binding. [file JCMM-29-e70416-s004.jpg]
